# Phenotyping, genome‐wide dissection, and prediction of maize root architecture for temperate adaptability

**DOI:** 10.1002/imt2.70015

**Published:** 2025-03-13

**Authors:** Weijun Guo, Fanhua Wang, Jianyue Lv, Jia Yu, Yue Wu, Hada Wuriyanghan, Liang Le, Li Pu

**Affiliations:** ^1^ Biotechnology Research Institute Chinese Academy of Agricultural Sciences Beijing China; ^2^ School of Life Science Inner Mongolia University Hohhot China; ^3^ College of Life and Environmental Sciences Hangzhou Normal University Hangzhou China

**Keywords:** GWAS, machine learning, maize, prediction, Root System Architecture

## Abstract

Root System Architecture (RSA) plays an essential role in influencing maize yield by enhancing anchorage and nutrient uptake. Analyzing maize RSA dynamics holds potential for ideotype‐based breeding and prediction, given the limited understanding of the genetic basis of RSA in maize. Here, we obtained 16 root morphology‐related traits (R‐traits), 7 weight‐related traits (W‐traits), and 108 slice‐related microphenotypic traits (S‐traits) from the meristem, elongation, and mature zones by cross‐sectioning primary, crown, and lateral roots from 316 maize lines. Significant differences were observed in some root traits between tropical/subtropical and temperate lines, such as primary and total root diameters, root lengths, and root area. Additionally, root anatomy data were integrated with genome‐wide association study (GWAS) to elucidate the genetic architecture of complex root traits. GWAS identified 809 genes associated with R‐traits, 261 genes linked to W‐traits, and 2577 key genes related to 108 slice‐related traits. We confirm the function of a candidate gene, *fucosyltransferase5* (*FUT5*), in regulating root development and heat tolerance in maize. The different *FUT5* haplotypes found in tropical/subtropical and temperate lines are associated with primary root features and hold promising applications in molecular breeding. Furthermore, we performed machine learning prediction models of RSA using root slice traits, achieving high prediction accuracy. Collectively, our study offers a valuable tool for dissecting the genetic architecture of RSA, along with resources and predictive models beneficial for molecular design breeding and genetic enhancement.

## Highlights

- Root anatomical traits were analyzed in 316 maize lines, revealing significant differences between tropical/subtropical and temperate lines.
- Genome‐wide association study identified 3511 candidate genes for Root System Architecture (RSA).
- *Fucosyltransferase5* gene function was validated using EMS mutation, showing associations of different haplotypes with primary root traits.
- Machine learning models were developed using root slice traits, achieving high accuracy in predicting maize RSA.

## Graphical abstract

This study dissects the genetic architecture of maize Root System Architecture, identifying significant root trait differences between tropical/subtropical and temperate lines. Using genome‐wide association study, 3511 genes were linked to root morphology, weight, and slice traits. The candidate gene *fucosyltransferase5* was validated for its role in root development and heat tolerance. Machine learning models based on root slice traits achieved high prediction accuracy, offering robust tools for ideotype‐based molecular breeding and genetic enhancement of maize.

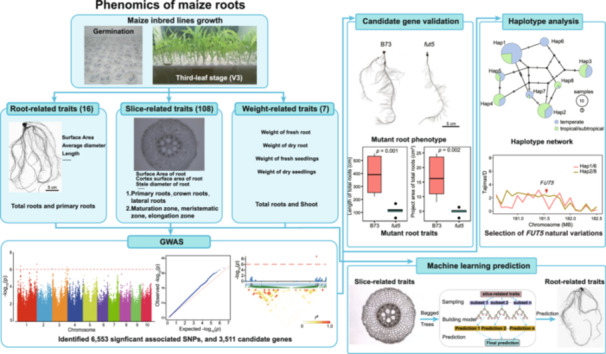

## INTRODUCTION

The diversity of maize root systems is evident in variations in rooting angles, the number of roots per type, and length and diameter within the root crown, which collectively define Root System Architecture (RSA) [1, 2]. RSA plays a crucial role in shaping essential agronomic traits, including plant anchorage, nutrient uptake, and stress responses, thereby serving as a pivotal factor in determining overall maize yield [3, 4]. Genetic enhancement of RSA is believed to contribute significantly to increased crop yields in high‐density planting systems [5]. However, observing and characterizing quantitative phenotypes of root systems is challenging due to the opacity of the root growth medium and the complexity of RSA [6]. Addressing these challenges necessitates interdisciplinary collaboration across fields such as mathematics, computer science, plant biology, and applied disciplines like plant breeding and agronomy [2, 7, 8].

The interest in high‐throughput root phenotyping platforms has been increasing among public and private research due to the labor‐intensive and destructive characteristics of traditional root measurement methods. Various high‐throughput automated root phenotyping platforms, including, Rhizoslides [9], Rhizoponics [10], GiA Roots [11], RootReader3D [12], and CPRS [13], have been developed to capture two‐dimensional (2D) or three‐dimensional (3D) images of roots grown in soil or non‐soil media. Based on these platforms, key genes regulating maize root structure, such as *ZmTIP1* (tip growth defective1) [14] and *ZmCIPK15* (calcineurin B‐like‐interacting protein kinase15) [15], which control root angle, have been identified. Ren et al. conducted root phenotyping of 14,301 field‐grown plants from an association mapping panel to study the genetic architecture of maize RSA and identified 81 high‐confidence RSA‐associated candidate genes, including *ZmRSA3.1* (Aux/IAA‐transcription factor 10) and *ZmRSA3.2*. Overexpression of *ZmRSA3.1* and *ZmRSA3.2* influences the maize auxin signaling pathway, altering auxin distribution in the crown root and ultimately modifying RSA [3]. However, methods for investigating root anatomy remain limited, restricting the discovery of genes associated with root microstructure.

Maize possesses five main root types: crown, seminal, primary, lateral, and brace roots [3, 16]. The majority of root biomass in mature maize plants originates from postembryonic roots that arise from the shoot. These postembryonic roots include crown roots, which develop below the soil surface, and brace roots, which emerge above the soil surface [16, 17]. The embryonic root system consists of primary and seminal roots, whose development is significantly influenced by genetic background [18, 19]. Lateral roots originate from the pericycle of other roots and significantly influence maize root architecture [20]. These root types play a crucial role in plant performance, as they are primarily responsible for water and nutrient uptake in maize. Several genes affecting maize root development have been identified, including *d1* (dwarf plant1), *d3* (dwarf plant3), *d5* (dwarf plant5), *BIGE1* (big embryo1), and *ZmHO‐1* (heme oxygenase 1). Mutations in *d1, d3, or d5* significantly increase the number of brace root whorls [21]. Loss‐of‐function mutations in *BIGE1* result in accelerated leaf and root initiation, an enlarged embryo scutellum, and an increased number of seminal and shoot‐borne roots [22]. *ZmHO‐1* in maize regulates lateral root development [23]. Although these genes have been identified, many loci affecting root growth and development remain unknown.

Except for brace roots, most maize roots grow underground, which makes detecting RSA challenging. Nevertheless, real‐time observation of RSA is essential for effective management of maize field. Machine learning, advanced through big data technologies and high‐performance computing [24, 25], enables the prediction of complex RSA traits by integrating high‐dimensional phenotypic and genomic data, offering new opportunities to revolutionize crop improvement in agri‐technologies. Predictive models utilizing various computational methodologies have proven effective for data‐driven decision‐making in phenotype prediction [26, 27]. In a previous study, we developed machine learning‐based predictive models using high‐throughput phenotyping (HTP) data, specifically image‐based digital traits, to predict final plant height from the early stage, with prediction accuracy improving rapidly across developmental stages [28]. Thus, utilizing HTP data facilitates the prediction of complex traits in maize. In this study, we integrated root anatomical data from 316 maize inbred lines with genotyping data from a genome‐wide association study (GWAS) to elucidate the genetic architecture of complex root traits. GWAS identified 3511 genes associated with three types of RSA traits, which may play crucial roles in maintaining RSA. Functional analysis of the candidate gene *FUT5* (galactoside 2‐alpha‐l‐fucosyltransferase5) in maize root development, using an ethyl methane sulfonate (EMS) mutant, confirmed the GWAS findings. The distinct *FUT5* haplotypes identified in tropical/subtropical and temperate lines are linked to primary root traits. Furthermore, predictive models based on machine learning, utilizing slice‐related traits (S‐traits) derived from maize root slices, demonstrated high predictive accuracy for RSA. In conclusion, our findings demonstrate that integrating RSA phenomic data, GWAS, and predictive modeling offers a novel approach to dissect the genetic architecture of maize roots, providing valuable insights for designing breeding strategies and genetic improvement in maize.

## RESULTS

### Identification of root phenotypes in maize seedlings

**Figure 1:**
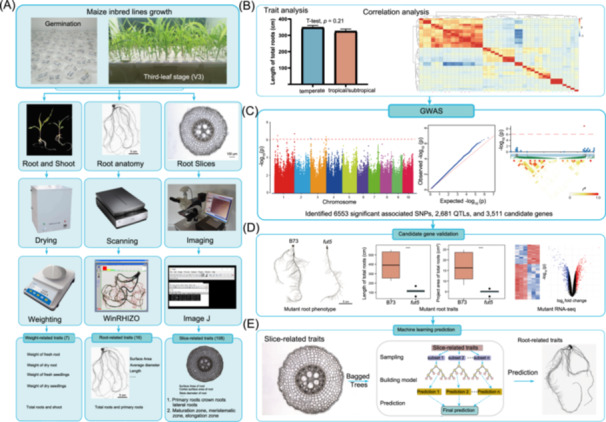
A flow diagram of the root architecture of maize was revealed by combining high‐throughput phenotypes, genome‐wide association study (GWAS), and predictive models. (A) Maize plant sowing, cultivation, and image acquisition in the greenhouse. Root‐related traits, slice‐related‐traits, and weight‐related traits were measured by different methods. Data processing from these three types of traits combined with correlation analysis (B), GWAS analysis (C), and functional validation of candidate genes (D). (E) A procedure showing machine learning‐based predictive models of RSA in maize. RSA, Root System Architecture.

RSA‐related traits are crucial for understanding how maize plants respond to various environmental conditions, including drought and nutrient uptake [29]. These traits play a key role in determining root growth patterns, nutrient acquisition efficiency, and overall plant vigor [30]. To obtain RSA‐related traits and cross‐sectional traits, we planted three‐leaf‐stage seedlings of 316 maize inbred lines and subsequently collected them (Figure 1A and Table S1). Sixteen root morphology traits (R‐traits), such as the length of total roots (Length), root volume of total roots (RootVolume), projected area of total roots (ProjArea), surface area of total roots (SurfArea), were analyzed using WinRhizo software. Full details are listed in Table S2. Additionally, seven plant weight‐related traits (W‐traits) were measured, including weight of fresh shoot (WFS), weight of dry shoot (WDS), weight of fresh root (WFR), weight of dry root (WDR), weight of fresh seedlings (WF), weight of dry seedlings (WD), and water content (WC). To observe the differences in root microphenotypes, we performed a cross‐sectional analysis of the meristematic, elongation, and maturation zones of primary, crown, and lateral roots. In total, 108 S‐traits were measured, with 12 S‐traits (such as diameter, surface area, and xylem number) for each of the three zones across three root types (Figure 1A, Table S2). The R‐traits, W‐traits, and S‐traits were further analyzed by correlation analysis and GWAS to reflect changes in root architecture (Figure 1B–E).

### Phenotypic variation of RSA traits in maize

**Figure 2:**
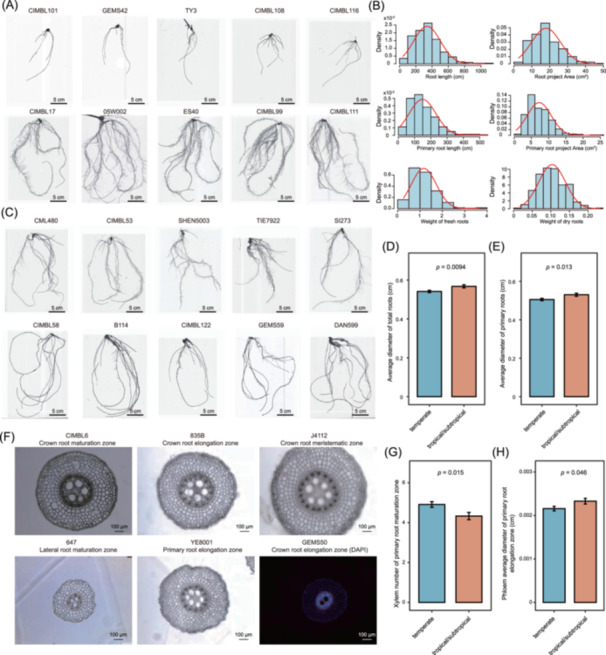
Root morphology and cross‐sectional phenotype analysis of different maize inbred lines. (A) Images of the roots at the three‐leaf stage in ten maize inbred lines, five with a sparse root structure and five with a dense root structure. (B) Distribution of R‐traits and W‐traits in 316 maize inbred lines. (C) Images of the roots at the three‐leaf stage in ten maize inbred lines, five with the thinnest root diameter and five with the thickest root diameter. Comparison of total root diameter (D) and primary root diameter (E) between tropical/subtropical and temperate maize inbred lines. (F) Cross‐sectional representative images of primary, crown, and lateral roots in maize. The blue image at the bottom right represents DAPI staining. Comparison of xylem number in the primary root maturation zone (G) and average phloem diameter in the primary root elongation zone (H) between tropical/subtropical and temperate maize inbred lines.

The root phenotypes of the 316 maize inbred lines exhibited significant natural variation, reflecting the diverse genetic backgrounds and environmental adaptations across the panel (Table S3). For instance, the CIMBL101, GEMS42, TY3, CIMBL108, and CIMBL116 inbred lines exhibited short roots with few branches. In contrast, the CIMBL17, 05W002, ES40, CIMBL99, and CIMBL111 inbred lines displayed long roots with numerous branches (Figure 2A). Frequency distribution analysis revealed that the lengths and projected areas of primary and total roots, as well as the fresh and dry root weights, followed a normal distribution (Figure 2B). Moreover, root diameter exhibited significant variation (Figure 2C). Notably, the primary and total root diameters of tropical/subtropical lines were greater than those of temperate lines (*p* = 0.0094 and 0.013, respectively) (Figure 2D,E), suggesting differences in RSA between tropical/subtropical and temperate lines. However, the total and primary root lengths of tropical/subtropical lines (325.3 ± 163.8 and 141.5 ± 90.2 cm, respectively) were slightly shorter compared to those of temperate lines (348.5 ± 161.8 and 155.1 ± 73.9 cm, respectively) (Figure S1). Furthermore, the projected area, number of tips, and number of crossings of primary roots in tropical/subtropical lines were significantly lower than those in temperate lines (*p* = 0.036, 0.033, and 0.024, respectively) (Figure S1). These results suggest that temperate maize inbred lines possess thinner, longer, and denser roots than tropical/subtropical maize, resulting in a significant increase in fresh seedling weight (*p* = 0.01) and water content (*p* = 0.015) (Figure S2).

Consistent with increased water content, the xylem number in the mature zones of primary roots in temperate maize lines was also greater than that in tropical/subtropical lines (*p* = 0.015) (Figure 2F,G). In the primary roots of temperate maize lines, the mature zone diameter and the xylem number in the elongation zone were greater, whereas the phloem diameter in the elongation zone was smaller (*p* = 0.046) (Figure 2H and Figure S3). However, in the lateral roots of temperate maize lines, the total diameter (*p* = 0.031), cortex diameter (*p* = 0.014), and phloem diameter (*p* = 0.031) of mature zones were significantly greater (Figure S4). Only the xylem number in the crown root mature zone of temperate lines was greater than that in tropical/subtropical lines (Figure S5). Moreover, no significant differences were observed in the diameter and area of the stele between temperate and tropical maize inbred lines across the three zones of the three root types (Figures S3–S5). These findings suggest that maize root architecture may have become slenderer during adaptation from tropical to temperate environments, potentially enhancing water uptake.

### Correlation analysis between root traits and plant architecture‐related traits

**Figure 3:**
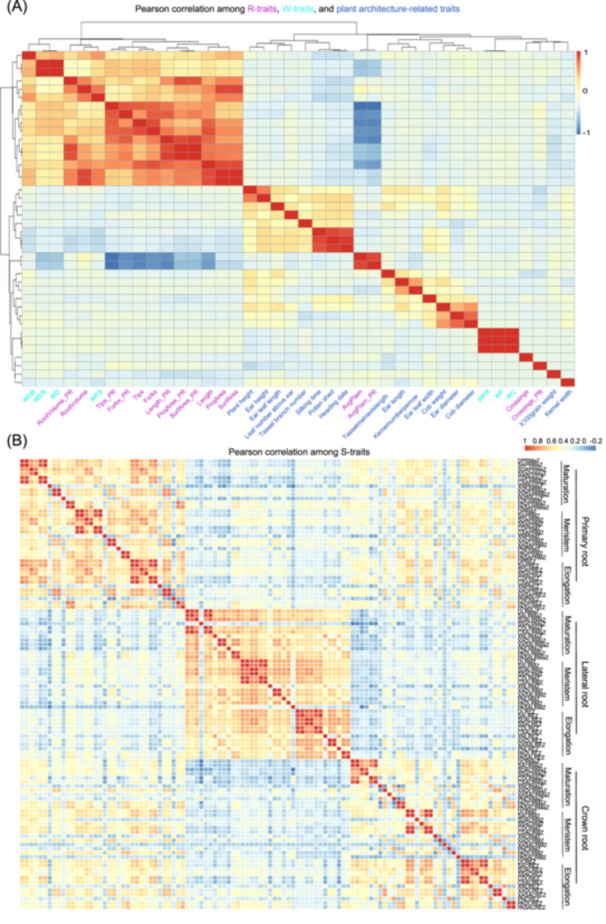
The relevance of the RSA and plant architecture‐related traits. (A) Heatmap displaying the correlation analysis among 16 R‐traits, 7 W‐traits, and plant architecture‐related traits. (B) Heatmap displaying the correlation analysis among 108 S‐traits. RSA, Root System Architecture.

Maize displayed positive yet low correlations between seedling and adult root traits [31]. To explore the relationship between seedling root traits and plant architecture‐related traits, we obtained 17 plant architecture‐related traits for the 316 maize inbred lines from the maizeGO database [32] and conducted a correlation analysis with our RSA traits. Among R‐traits, total root length showed positive correlations with key R‐traits and W‐traits, such as SurfArea (*R* = 0.90, *p* = 2.22 × 10^−16^), RootVolume (*R* = 0.72, *p* = 2.22 × 10^−16^) and WFS (*R* = 0.65, *p* = 2.22 × 10^−16^), and WDR (*R* = 0.49, *p* = 2.22 × 10^−16^), suggesting that root length affects both root and plant biomass (Figure 3A and Table S4). However, a negative correlation was observed between root length and AvgDiam, representing the average diameter of the total root (*R* = −0.44, *p* = 5.77 × 10^−15^), potentially explaining the long, thin roots observed in temperate maize (Figure 2D,E and Figure S1). Water content displayed a strong positive correlation with WFR (*R* = 0.999, *p* = 2.22 × 10^−16^) and WF (*R* = 0.999, *p* = 2.22 × 10^−16^) but no correlation with fresh shoot weight (*R* = 0.07) (Figure 3A and Table S4). Furthermore, most RSA‐related traits and plant architecture‐related traits showed low or no significant correlations (*R* = −0.20–0.11) (Table S4), which may be attributed to the differences in developmental stages and growing conditions between the two datasets. Nevertheless, we identified a low negative correlation between 100‐kernel weight and certain root traits, including total root length (*R* = −0.135, *p* = 0.021), tips (*R* = −0.116, *p* = 0.047), and primary root length (*R* = −0.176, *p* = 0.002) (Table S4).

For S‐traits, positive correlations were observed between traits from primary, lateral, and crown roots, as well as within each root type (Figure 3B). Specifically, S‐traits from primary roots showed positive correlations with those from crown roots but negative correlations with traits from lateral roots (Figure 3B), suggesting differences between primary and lateral roots. Furthermore, S‐traits from primary roots were positively correlated with key R‐traits of primary roots (Figure S6). In summary, W‐traits, R‐traits, and S‐traits were correlated to some extent and could mutually influence each other in determining the total biomass of maize. These findings suggest that these traits could be utilized for predicting RSA.

### Genetic basis of root architecture in maize

**Figure 4:**
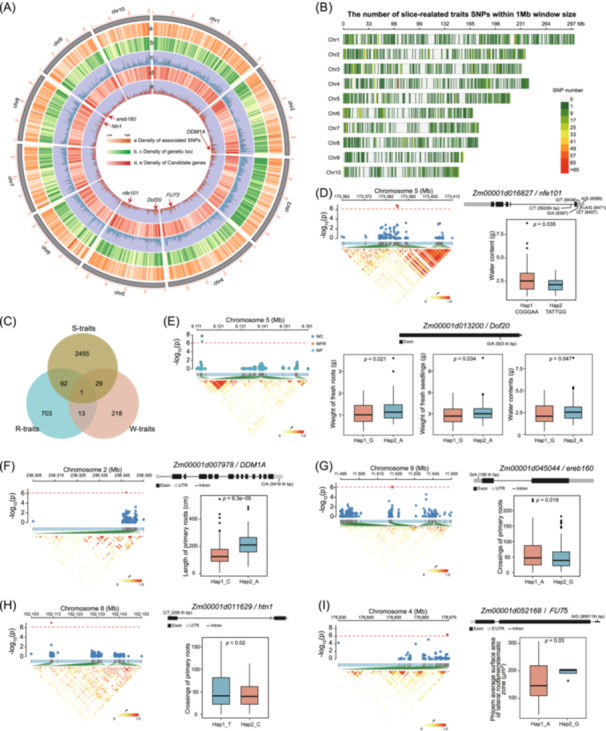
GWAS identification of candidate genes for variation in W‐traits, R‐traits, and S‐traits in maize. (A) Chromosomal distribution of loci associated with all traits. Each vertical line indicates a lead SNP or locus. (B) Chromosomal distribution of loci associated with s‐related traits. Each vertical line indicates a lead SNP. (C) Venn diagram displaying the number of loci associated with W‐traits, R‐traits, and S‐traits. (D) Local Manhattan plot and LD statistic *r* ^2^ values (left) for the *nef101* gene associated with a W‐trait, WC. The upper right panel displays the gene structure of *nef101*. The distributions of water content for the two groups based on 6 SNPs are shown in the boxplot. (E) Local Manhattan plot and LD statistic *r* ^2^ values (left) for the *Dof20* gene associated with three W‐traits, WFR, WF, and WC. The upper right panel displays the gene structure of *Dof20*. The distributions of WC, WFR, and WF for the two groups based on the SNP chr5.S_6113770 are shown in the boxplot. (F) Local Manhattan plot and LD statistic *r* ^2^ values (left) for the *DDM1A* gene associated with a R‐traits, Length_PR. The upper right panel displays the gene structure of *DDM1A*. The distributions of Length_PR for the two groups based on the SNP chr2.S_236352566 are shown in the boxplot. Local Manhattan plot and LD statistic *r* ^2^ values (left) for the genes *ereb160* (G) and *htn1* (H) associated with a R‐traits, Crossings_PR. The upper right panel displays the gene structure of *ereb160* (G) and *htn1* (H). The distributions of Crossings_PR for the two groups based on the SNPs chr9.S_11532992 (G) and chr8.S_152112334 (H) are shown in the boxplot. (I) Local Manhattan plot and LD statistic *r* ^2^ values (left) for the *FUT5* gene associated with a S‐traits, Phloem average surface area of lateral root meristematic zone (PASALRMZ). The upper right panel displays the gene structure of *FUT5*. The distributions of PASALRMZ for the two groups based on the SNP chr4.S_178676036 are shown in the boxplot.

To investigate the genetic and molecular basis of W‐traits, R‐traits, and S‐traits, we performed a GWAS analysis to analyze genomic variants in the association panel. This analysis identified 6554 trait‐associated SNPs, including 4397 unique SNPs, of which 831 were associated with R‐traits, 384 with W‐traits, and 3182 with S‐traits (Figure 4A,B, Figure S7A,B, and Table S5). We then identified candidate genes linked to significant SNPs. A total of 3511 unique candidate genes associated with 4,397 significant associations were identified and annotated (Table S5). Each SNP was associated with an average of 1.49 traits (ranging from 1 to 7) (Figure S7C and Table S6). We defined SNPs with ≥6 associations as hub SNPs based on 5% of the total number of SNPs [28], and identified 339 hub SNPs (Figure S7C). A Venn diagram showed that only one unique gene, *Zm00001d052170*, which encodes a fucosyltransferase, was associated with all three trait types (Figure 4C, Table S6). Notably, 261 genes associated with W‐traits were enriched in GO terms such as Carbohydrate metabolic process and polysaccharide metabolic process (Figure S7D), of which 11.4% (30/261) overlapped with S‐traits (Figure 4C). Additionally, 669 genes were linked to R‐traits, 11.4% (93/809) of which also overlapped with S‐traits (Figure 4C). These 669 genes were enriched in GO terms such as developmental process, regulation of anatomical structure morphogenesis, and biosynthetic process. Similarly, the 2577 genes linked to S‐traits were enriched in GO terms related to root development and function, including defense response to bacterium, regulation of developmental growth, and amino acid biosynthetic process (Figure S7D). Notably, we cross‐referenced the genes identified by GWAS with those specifically associated with roots, as compiled in a previous study [33], and discovered 73 genes that are specific to roots (*p* = 8.9 × 10^−4^) (Figure S8A). These genes are likely involved in key processes such as root elongation, branching, and nutrient uptake, which are essential for maize RSA and overall plant performance [33]. These 73 genes were enriched in GO terms related to growth and flower development, response to endogenous stimulus, cell differentiation, and signal transduction (Figure S8B). The set of 73 genes included *Zm00001d027925* (AP2‐EREBP‐transcription factor 180, *ereb180*) [34], *Zm00001d030121* (roothairless6, *rth6*) [35], *Zm00001d034383* (Viviparous8, *vp8*) [36], *Zm00001d042961* (Respiratory burst oxidase1, *rboh1*, also called *rth5*) [37], *Zm00001d018024* (PIN‐formed protein2, *pin2*) [38], *Zm00001d04481*(PIN‐formed protein1, *pin1*) [38], *Zm00001d024909* (CO CO‐LIKE TIMING OF CAB1 protein domain1, *cct1*) [39], all of which have been previously validated for their roles in influencing root development and stress response (Table S6). Furthermore, we compared the previous RSA GWAS study with the 297 genes identified in earlier GWAS results [40, 41] (Figure S8C). These findings confirm the reliability of our GWAS results, providing a valuable pool of genetic resources for investigating maize root development and breeding.

### GWAS for W‐traits, R‐traits, and S‐traits

Among the 261 genes associated with 7 W‐traits, the highest number of genes was associated with WFR and WC, totaling 213 and 81, respectively (Table S6). The gene *Zm00001d016827* (nucleosome/chromatin assembly factor group E 101, *nef101*) contains six significant SNPs (*p* < 7.97 × 10^−7^) associated with three W‐traits: WC, WFR, and WF (Figure 4D and Table S6). Plants carrying the Hap2 (TATTGG) allele, based on these six SNPs, exhibited significantly lower water content (Figure 4D). *Zm00001d013200* encodes the C2C2‐Dof‐transcription factor 24 (*Dof24*), which contains a DOF domain and may play a vital role in plant growth and development, including signal transduction, morphogenesis, and resistance to environmental stress [42]. A mutation in *Dof24* at chr5.S_6113770 was associated with WFR (*p* = 2.00 × 10^−8^), WF (*p* = 3.30 × 10^−7^), and WC (*p* = 4.59 × 10^−7^) (Figure 4E and Table S6). This SNP, located within the gene body of *Dof24*, results in a missense variant. Plants with the A allele at position 923 of *Dof24* exhibited significant increases in WFR, WF, and WC (*p* = 0.021, 0.034, 0.047, respectively) (Figure 4E). These results indicate a potential role for *nef101 and Dof24* in regulating water and nutrient uptake in maize roots.

A total of 831 unique SNPs associated with R‐traits, corresponding to 809 candidate genes, were identified (Table S5). Several candidate genes were involved in DNA‐templated transcription (Figure S7D). For example, we identified one SNP (chr2.S_236352566) significantly associated with primary root length (*p* = 6.28 × 10^−^^7^) (Table S6). This SNP has two natural alleles in the 3′‐UTR of gene *Zm00001d007978* (decrease in DNA methylation 1a, *DDM1A*), and plants carrying the A allele exhibited longer primary root length (*p* = 9.3 × 10^−^^5^) (Figure 4F). It has been reported that *ddm1a* mutants exhibits a significantly higher average root diameter index and a significantly lower root length index compared to the wild type [43]. *DDM1A* influences root length through epigenetic regulation, which is consistent with its known role in early embryo development [43], thereby supporting our GWAS results.

The gene e*reb160* is homologous to rice *OsERF71*, a transcription factor known to regulate root structure and enhance drought resistance in rice when overexpressed [44]. The SNP chr9.S_11532992, located in the 5′‐UTR of the *ereb160* gene, results in a start codon gain variant when mutated to the A allele. This SNP was associated with the R‐trait crossings of the primary root (*p* = 7.74×10^−7^), where plants carrying the A allele exhibited more primary root crossings (*p* = 0.019) (Figure 4G). These findings suggest that *ereb160* may influence root architecture traits through transcriptional regulation, contributing to root system adaptability. Another SNP, chr9.S_11532641 in the *ereb160* gene, was associated with WFR (Figure S9), further supporting its potential role in regulating RSA and water uptake traits.

In addition, plants carrying the *Htn1* (*Helminthosporium turcicum* resistance N1) gene exhibited a transition between lesion‐free status and the presence of a few wilt‐type lesions, resembling small soak spots [45]. The maize disease resistance gene *Htn1*, which confers resistance to northern corn leaf blight, encodes a wall‐associated receptor‐like kinase [46]. The SNP chr8.S_152112334, carrying the T allele, resulted in a missense variant in the *Htn1* gene and was associated with an increased number of primary root crossings (*p* = 1.09 × 10^−^^7^) (Figure 4H). *Zm00001d052168* (*FUT5*) and *Zm00001d052170* encode a fucosyltransferase, with *Zm00001d052170* being the only gene associated with all three trait types (Figure 4I and Table S6). Notably, both *FUT5* and *Zm00001d052170* may be linked to the s‐trait phloem average surface area of the lateral root meristematic zone (*p* = 7.29 × 10^−^^7^) (Figure 4I and Table S6). In summary, these findings suggest that the root gene mutations identified by GWAs may play a crucial role in the development and function of maize roots.

### Functional verification of the candidate root architecture gene *FUT5*

**Figure 5:**
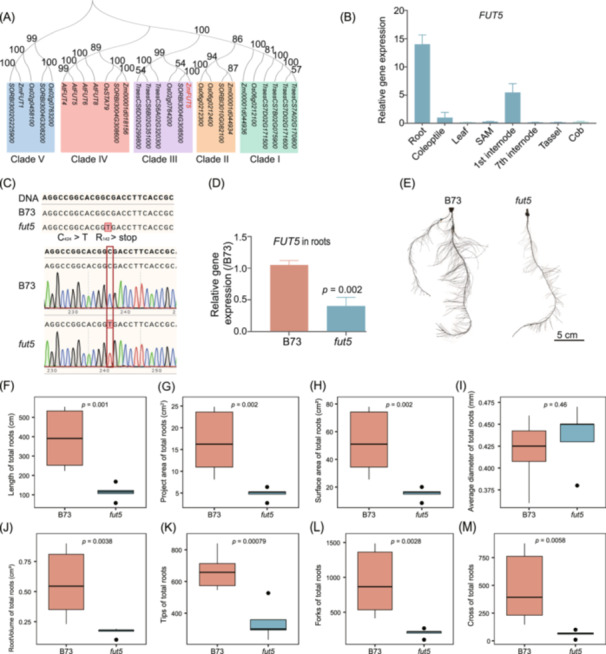
Effects of the *fucosyltransferase5* (*FUT5*) gene on maize RSA. (A) Phylogenetic tree of the *FUT5* gene among Arabidopsis, rice, maize, soybean, and wheat. (B) The expression level of *FUT5* gene in different maize tissues. SAM, shoot apical meristem. (C) Sequence alignment (partial) of the *FUT5* gene in B73 wild‐type and *fut5* mutant plants. The red box marks the mutation site in the *fut5* mutant. (D) The expression level of *FUT5* gene in the *fut5* mutant. (E) Root phenotype of *fut5* mutant. Comparative analysis of 8 root traits between the *fut5* mutant and B73 maize roots, including total root length (F), root area (G), root surface area (H), root diameter (I), root volume (J), root tips (K), root forks (L), and crossings of total roots (M).

To validate the impact of gene mutations identified through GWAS on maize root development and function, we investigated the role of the maize *fut5* mutant. The *AtFUT* family in *Arabidopsis*, comprising at least 10 genes, likely includes fucosyltransferases essential for wall carbohydrate synthesis [47]. Phylogenetic analysis revealed that *ZmFUT5* shares high homology with *Os02g0764200* in rice, as well as *AtFUT4, AtFUT5, AtFUT6*, and *AtFUT8* in *Arabidopsis* (Figure 5A). *AtFUT4* and *AtFUT6*, both fucosyltransferases specific to arabinogalactan proteins, exhibit distinct expression patterns in roots and leaves, as well as different sub‐localization in roots [48], suggesting that *ZmFUT5* may have similar functions in regulating root development. Interestingly, *ZmFUT5* exhibits the highest gene expression in roots but is not expressed in the cob, tassel, and leaf (Figure 5B). To validate the role of *FUT5* in modulating maize root architecture, we obtained a B73 EMS mutant (*fut5*, Mut_sample EMS4‐270411) from a maize EMS mutant library (http://maizeems.qlnu.edu.cn/). This mutant harbors a C‐to‐T base mutation at position 424, resulting in an early stop codon at amino acid position 142 (Figure 5C). Quantitative reverse‐transcription PCR (qRT–PCR) confirmed reduced *FUT5* expression in the roots of *fut5* mutants (Figure 5D). The roots of *fut5* mutants exhibited distinct RSA compared to wild‐type B73 roots (Figure 5E). The *fut5* mutation led to reductions in root length (*p* = 1.0 × 10^−^^3^), projected area (*p* = 2.0 × 10^−3^), surface area (*p* = 2.0 × 10^−^^3^), root volume (*p* = 3.8 × 10^−^^3^), number of tips (*p* = 7.9 × 10^−^^4^), forks (*p* = 2.8 × 10^−^^3^), and crossings (*p* = 5.8 × 10^−^^3^) of total roots, while the average diameter remained unchanged (*p* = 0.46) compared to B73 roots (Figure 5F–M). These results highlight the essential role of *FUT5* in regulating maize RSA and its potential involvement in root development.

**Figure 6:**
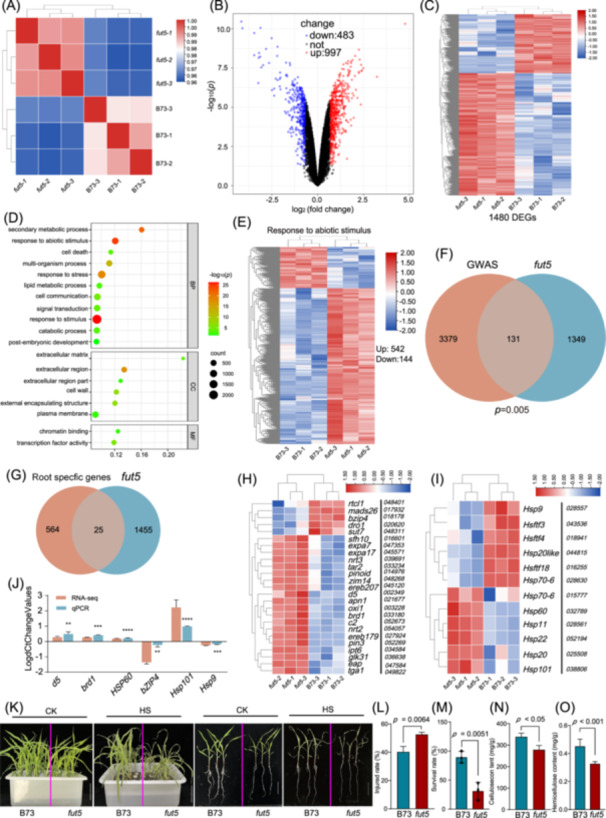
Transcriptome analysis of differentially expressed genes (DEGs) regulated by the *fucosyltransferase5* (*FUT5*) gene. (A) Pearson's correlation analysis of gene expression in roots between *fut5* mutants and B73 seedlings. Volcano plot (B) and Heatmap (C) showing the number of DEGs regulated by the *FUT5* gene. (D) Functional enrichment of *fut5*/B73‐specific DEGs. (E) Heatmap illustrating genes implicated in the regulation of responses to abiotic stimulus. (F) A Venn diagram displays the unique and shared genes between the DEGs of *fut5* mutants and the genes identified by GWAS for RSA. (G) A Venn diagram displays the unique and shared genes between the DEGs of *fut5* mutants and the root‐specific genes summarized by the previous study [33]. Heatmap illustrating 25 root‐specific DEGs (H) and 12 heat stress (HS)‐related DEGs (I). The number represents the B73 RefGen_V4 gene name. (J) Quantitative PCR results of selected root specific and HS‐related genes. Statistical significance was tested using a two‐tailed *t*‐test (mean ± SD; **p < 0.05, ***p < 0.001, ****p < 0.0001). The phenotype (K), heat injury ratio (L), and survival rate (M) of *fut5* mutants treated by HS were analyzed. Bar = 10 cm. Cellulose (N) and hemicellulose content (O) were measured in *fut5* mutants and WT plants. GWA, genome‐wide association study; RSA, Root System Architecture.

To explore the molecular mechanisms underlying RSA regulation by *FUT5*, we conducted RNA‐seq analysis on both *fut5* mutants and B73 plants. Fragments per kilobase per million mapped reads (FPKM) values for all maize genes were used to construct a Pearson correlation matrix to compare the transcriptomes of *fut5* and B73. The heatmap revealed a clear distinction between *fut5* and B73 groups, with high correlation among the three biological replicates (Figure 6A). From the RNA‐seq data, we identified 997 upregulated and 483 downregulated differentially expressed genes (DEGs) with |log2 fold change | > 1 and false discovery rate < 5%, indicating more upregulated DEGs than downregulated DEGs (Figure 6B,C). GO analysis revealed that DEGs were primarily associated with processes such as response to stimulus, secondary metabolic processes, cell death, and lipid metabolic processes under the molecular function category. These DEGs were located in the extracellular matrix, cell wall, and plasma membrane under the cellular component category (Figure 6D). Additionally, at least 542 upregulated and 144 downregulated DEGs were involved in response to abiotic stimulus (Figure 6E). Approximately 8.85% (131 out of 1480, *p* = 0.005) of the DEGs regulated by *FUT5* overlapped with genes identified in our GWAS results (Figure 6F), while 25 DEGs (Figure 6G) were identified as root‐specific genes in a previous study [33]. The 25 DEGs include *Zm00001d048401* (*RTCS‐like1*, *rtcl1*), *Zm00001d017932 (MADS‐transcription factor 26, mads26)*, *Zm00001d033180* (*Brassinosteroid‐deficient dwarf1*, *brd1*), *Zm00001d018178* (*bZIP‐transcription factor 4*, *bzip4*), *Zm00001d002349* (*Kaurene synthase3*, *ks3*, also known as *d5*), and *Zm00001d049822* (*Teosinte glume architecture1*, *tga1*), all of which are essential for root development [21, 49, 50, 51, 52, 53] (Figure 6H). The qPCR results confirmed downregulation of *bzip4* in *fut5* mutants, whereas *d5* and *brd1* were upregulated (Figure 6I), suggesting the potential involvement of these genes in the regulatory role of *FUT5* on RSA.

Additionally, mRNA expression of several heat shock protein (HSP)‐related genes or heat shock transcription factor (Hsftf)‐related genes was altered in *fut5* mutants (Figure 6J). For example, *Hsp9* was downregulated, whereas *Hsp60* and *Hsp101* were upregulated, as confirmed by qPCR (Figure 6I), indicating an impact on heat tolerance in *fut5* mutants. Subsequently, we subjected *fut5* and B73 seedlings to heat stress and observed that *fut5* plants exhibited lower heat tolerance and a higher heat stress damage ratio compared to the B73 plants (Figure 6K–M). As a galactoside 2‐alpha‐l‐fucosyltransferase, FUT5 protein plays a crucial role in hemicellulose biosynthesis [54]. Both cellulose and hemicellulose content were significantly decreased in *fut5* mutants (Figure 6N,O). RNA‐seq data revealed that *FUT5* downregulation affected genes related to stress responses and cell wall metabolism, consistent with its observed phenotypic effects (Figure 6K). These results validated the reliability of transcriptome sequencing in *fut5* mutants and highlighted the significant role of *FUT5* in root development and response to abiotic stress. In summary, our findings underscore the crucial role of *FUT5* in regulating RSA‐related traits, emphasizing the predictive potential of key genes identified in our analysis for optimizing RSA breeding in maize.

### Selection of *FUT5* natural variations during the spread of maize from tropical to temperate regions

**Figure 7:**
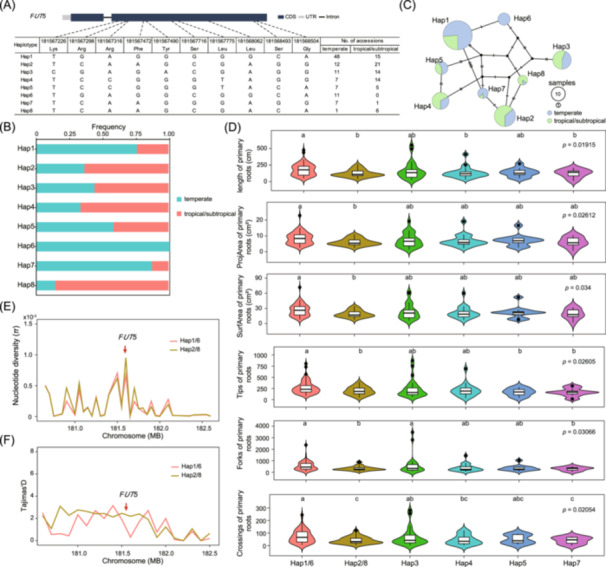
Selection of *fucosyltransferase5* (*FUT5*) natural variations during the spread of maize from tropical to temperate regions. (A) Haplotypes of *FUT5* amongst maize natural variations. (B) Distribution frequency of *FUT5* haplotypes in tropical/subtropical and temperate maize inbred lines. (C) A haplotype network showing the eight major haplotypes in tropical/subtropical and temperate maize inbred lines. (D) Root length, total area, surface area, tips, forks, and crossings of primary roots amongst haplotype groups. The phenotype distribution of each haplotype group is displayed by the vioplot. Multiple comparisons of the trait values were made by the least significant difference (LSD) method. Statistical significances at *p* < 0.05 are indicated with different lowercase letters. Nucleotide diversity (*π*) (E) and Tajima's *D* (F) value at *FUT5* gene amongst haplotype groups.

To gain deeper insights into the evolution of *FUT5* natural variations, we examined genetic variation in *FUT5* among tropical/subtropical and temperate maize inbred lines. Across tropical/subtropical and temperate lines, 8 haplotypes were identified in the coding region of *FUT5* (Figure 7A). Haplotypes Hap1 and Hap6, enriched with temperate lines (Figure 7B,C), comprising 66 of 82 temperate lines. In contrast, Hap2 and Hap8, enriched in tropical/subtropical lines (Figure 7B,C), comprising 27 of 40 tropical/subtropical lines. After consolidating Hap1 with Hap6, and Hap2 with Hap8 due to their similar DNA sequences, we examined associations between genetic variation in *FUT5* and root‐related traits in 316 maize accessions. The analysis included primary root length (*p* = 0.019), area (*p* = 0.026), surface area (*p* = 0.034), tips (*p* = 0.026), forks (*p* = 0.03), and crossings (*p* = 0.02) among different haplotypes. The results revealed significant differences in these traits among different haplotypes (Figure 7D). Furthermore, the six R‐traits in Hap1/6, including increased root area, surface area, tips, and crossings, were significantly higher than those in Hap2/8 (Figure 7D and Figure S1), potentially enhancing water‐use efficiency in temperate maize. Therefore, we hypothesized that *FUT5* may have been a target of selection during maize adaptation to different regions. To test this hypothesis, we estimated nucleotide diversity (*π*) and Tajima's *D* values for the *FUT5* CDS region among haplotype groups. The result showed that the nucleotide diversity (*π*) in the *FUT5* region with Hap1/6 haplotypes was lower than that of Hap2/8 haplotypes (Figure 7E). Tajima's *D* values for the *FUT5* region in Hap1/6 were approximately 0.51, compared to 2.16 in Hap2/8 haplotypes (Figure 7F). These results indicate positive selection acting on the *FUT5* gene. Collectively, these findings highlight the critical role of *FUT5* in driving maize adaptation to diverse environmental conditions, particularly during its spread from tropical to temperate regions.

### Machine learning‐based predictive models for root architecture using cross‐section traits

**Figure 8:**
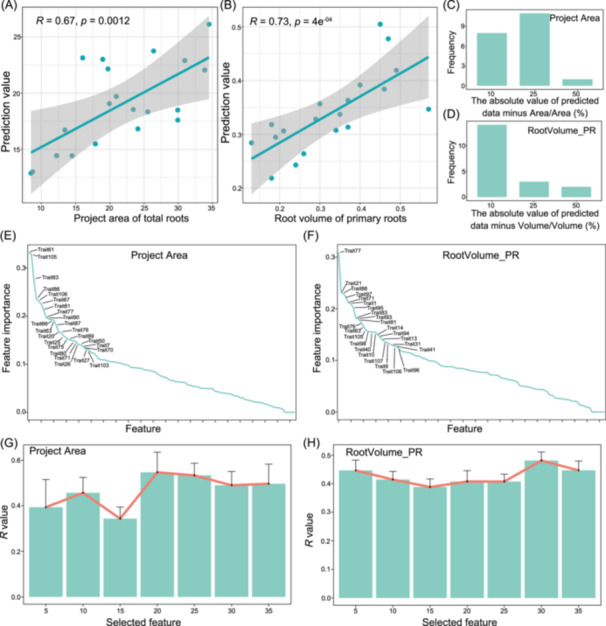
Prediction of R‐traits using S‐traits. Prediction accuracies of total root area (A) and primary root volume (B) using all S‐traits. Relative error of total root area (C) and primary root volume (D) prediction in maize accessions. The formula is (predicted R‐traits minus R‐traits)/R‐traits × 100%. Importance of S‐traits for total root area (E) and primary root volume (F) prediction using a random forest model. Prediction accuracies of total root area (G) and primary root volume (H) using all S‐traits or selected S‐traits with top‐5 to top‐35 S‐trait importance.

In the field of plant Big Data analytics, machine learning plays a pivotal role, autonomously identifying data patterns, optimizing model parameters, and uncovering intricate genetic and environmental factors underlying complex plant traits [28, 55]. Due to the opacity of the root growth medium and the complexity of RSA, observing and quantifying root phenotypes is challenging. The S‐traits were easier to collect than the whole R‐traits, representing a portion of the root system, and could be advantageous for predicting entire R‐traits in maize using machine learning. We assessed the predictive performance of R‐traits based on S‐traits using 10‐fold cross‐validation with a bagging tree model, which has demonstrated effectiveness in previous studies involving machine learning and remote sensing [56, 57]. The correlation coefficients between predicted and true values for ProjArea and RootVolume_PR were 0.67 (*p* = 1.2 × 10^−3^) and 0.73 (*p* = 4.0 × 10^−4^), respectively (Figure 8A,B). Furthermore, the relative error was less than 25% for ProjArea of most maize accessions and less than 10% for RootVolume_PR (Figure 8C,D). These findings suggest that utilizing S‐traits to predict root area and volume could serve as a promising robotic approach for optimizing RSA selection and crop breeding.

To identify key determinants of root area and volume in predictive models among S‐traits, we iteratively selected features based on their importance in the comprehensive machine‐learning model. Among the top 35 S‐traits contributing to root area, key contributors included trait61 (Diameter of lateral roots elongation zone) and trait105 (Xylem average surface area of crown roots elongation zone), which are critical for nutrient and water uptake efficiency (Figure 8E). For root volume, significant traits included trait77 (Cortex diameter of crown roots differentiation zone), and trait88 (Stele surface area of crown roots meristematic zone), reflecting their roles in root structural integrity and transport capacity (Figure 8F). We then selected varying numbers of S‐traits based on feature importance to validate the predictive models. To achieve a prediction accuracy above 0.4 for root area, more than 20 S‐traits were required (Figure 8G), whereas for root volume, only 5 S‐traits were needed to achieve the same level of accuracy (Figure 8H). These results suggest that S‐traits of crown and lateral roots are crucial for predicting root area, whereas S‐traits of primary, crown, and lateral roots all play significant roles in predicting root volume. The bagging tree model demonstrated that RSA could be partially predicted using only five S‐traits from root phenotypic data, highlighting its potential as an efficient tool for HTP in breeding programs. By focusing on key S‐traits, this approach may facilitate the rapid identification of desirable root traits, accelerating the selection process in maize breeding.

## DISCUSSION

This study integrates phenotypic and genomic data with machine learning to dissect the genetic architecture of RSA traits, providing novel insights into maize adaptation. By analyzing 316 maize inbred lines, we collected 16 R‐traits, 7 W‐traits, and 108 S‐traits and identified 6554 trait‐associated SNPs, including 4397 unique SNPs, through GWAS. Among these, 831 unique SNPs were associated with R‐traits, 384 with W‐traits, and 3182 with S‐traits, highlighting their potential roles in maintaining RSA (Table S5). The plastic and dynamic nature of plant RSA enables adaptation for optimal soil resource acquisition, and our findings emphasize the potential for productivity gains through RSA optimization, particularly under low‐input conditions [58]. Although conventional QTL mapping of RSA in maize has been performed [17, 40, 59, 60, 61], this study represents one of the first comprehensive efforts to genetically map the complete anatomy and structure of multiple root systems in a natural diversity maize panel (Figure 1).

RSA variations reflect maize adaptation to temperate and tropical environments, emphasizing their role in environmental resilience [62]. A previous study suggested that maize inbred lines from tropical/subtropical regions exhibit shorter root hairs, indicating differences in RSA between tropical/subtropical and temperate maize lines [63]. Our findings revealed that tropical/subtropical lines had slightly shorter total and primary root lengths compared to temperate lines. Moreover, the projected area, thenumber of tips, and crossings of primary roots were significantly reduced in tropical/subtropical lines compared to temperate lines (Figure S1). However, the primary and total root diameters of tropical/subtropical lines were greater than those in temperate lines (Figure 2D,E). In contrast, a previous study reported that the median bracing root diameter in temperate lines was significantly greater than that in tropical/subtropical lines [61]. This discrepancy may be due to differences in maize genotypes, developmental stages, and root types tested. RSA plays a vital role in plants, such as facilitating soil water uptake‐a crucial process during droughts and challenging under waterlogging conditions [64]. Notably, temperate maize inbred lines exhibited a significant increase in fresh seedling weight and water content (Figure S2), which is associated with a higher xylem number in the primary root compared to tropical/subtropical lines (Figure 2F,G). These results underscore the critical changes in RSA that facilitated maize adaptation from tropical to temperate zones, supporting environmental resilience during its expansion [40, 62].

Maize domestication and adaptation to diverse environmental conditions have driven changes in numerous genes through directional selection [40]. Despite extensive genetic studies on maize root traits [15, 33, 41, 65, 66, 67], few genes associated with root anatomical traits have been identified and functionally characterized. In this study, we identified numerous genes with small to moderate phenotypic effects (Figure 4A,B, Table S6), reflecting the genetic complexity of RSA and root anatomical traits. Many of these genes are root‐specific (Figure S8A) [33], highlighting their specialized roles in root development. Among these, *Dof24*, a transcription factor with a DOF domain critical for plant growth and development [42], was significantly associated with WFR, WF, and WC (Figure 4E, Table S6). Its rice homolog *OsDOF15*, positively regulates primary root elongation by controlling cell proliferation in the root meristem and limiting ethylene biosynthesis [68]. This functional evidence suggests that *Dof24* may influence maize RSA by regulating cell proliferation in root meristems, thereby enhancing water and nutrient uptake efficiency. These findings provide critical insights into the genetic regulation of maize root traits and underscore the potential of *Dof24* as a target for RSA optimization in breeding programs.

*DDM1A* is involved in the establishment and maintenance of proper DNA methylation [69]. In our study, a mutant of this gene was linked to primary root length (Figure 4F). A previous study reported that *DDM1A* mutants had considerably lower shoot and root biomass under normal‐Pi conditions, whereas they exhibited significantly greater shoot and root biomass under low‐Pi stress [43]. The maize genome encodes two genes, *DDM1A* and *DDM1B*, which are homologous to Arabidopsis *DDM1* and are likely retained duplicates from the most recent whole‐genome duplication event [69]. Simultaneous disruption of the two *ZmDDM1* homologs, which are abundantly expressed in the embryos, led to embryo lethality and abnormalities in cell proliferation during early kernel development. This regulation occurs through the formation of ^m^CHH islands via the RdDM pathway in maize [70]. In summary, *DDM1A*, as a chromatin remodeler involved in histone modifications and DNA methylation, plays an important role in maize shoot and root development.

A previous study indicated that *FUT5* is predominantly expressed in the root tips and meristem zones of maize [71]. Our results further demonstrated that the roots of *fut5* mutants exhibited distinct RSA compared to wild‐type (Figure 5E), likely due to the specific high expression of *FUT5* in maize roots (Figure 5B). As a galactoside 2‐alpha‐l‐fucosyltransferase, the FUT5 protein is critical for adding terminal fucosyl residues to xyloglucan side chains, a key step in cell wall biosynthesis [54]. RNA‐seq data revealed significant alterations in the expression of cell wall‐related genes in *fut5* mutants, including a substantial number of DEGs involved in cell wall metabolism (Figure 6D), which confirms FUT5's essential role in cell wall synthesis. Notably, *bzip4* was downregulated in *fut5* mutants, whereas *d5* and *brd1* were upregulated (Figure 6H,I), all of which are key regulators of root development [21, 50, 51]. Interestingly, mutations in *FUT5* led to numerous DEGs associated with abiotic stress (Figure 6D,J). Heat shock experiments further validated the sensitivity of *fut5* mutants to high temperatures (Figure 6K,L). This sensitivity may be attributed to the reduced cellulose and hemicellulose levels observed in *fut5* mutants (Figure 6M,N), as these compounds are crucial for maintaining cell wall integrity and protecting plants from heat‐induced damage. Previous studies have shown that cellulose and hemicellulose contribute to cell wall rigidity and thermal stability, enabling plants to withstand high temperatures by preserving cellular structure under stress conditions [72, 73].

Moreover, measuring most RSA traits in the field using traditional methods is challenging due to the subterranean nature of maize roots. Our previous study showed that machine learning‐based predictive models using HTP data to predict final plant height from the early stage were available [28]. Therefore, root S‐traits, obtained by sampling portions of the root system, offer a feasible approach for predicting the entire RSA through machine learning. In this study, we developed a predictive model and conducted a series of assessments on S‐traits to evaluate their influence on the accuracy of maize r‐trait prediction. A strong correlation between predicted and actual values for root area and primary root volume was achieved using S‐traits (Figure 8A,B). Furthermore, the S‐traits from crown and lateral roots are essential for predicting root area, while S‐traits of primary, crown, and lateral roots collectively contribute significantly to predicting root volume. Notably, predicting root volume with the same level of accuracy required only five S‐traits (Figure 8H). Thus, our findings demonstrate the potential of machine learning‐based predictive models for root traits using S‐traits, providing a theoretical foundation for data‐driven phenotyping to accelerate crop breeding.

## CONCLUSION

In summary, the integration of root phenotyping, GWAS, and predictive modeling have elucidated the genetic architecture underlying RSA for temperate adaptability in maize, offering valuable insights into optimizing root traits for maize breeding.

## METHODS

### Plant materials and experiment design

The 316 maize inbred lines, detailed in Table S1, were used for evaluating root traits and conducting GWAS, as described in a prior publication [28]. Maize seeds were surface sterilized with a 10% H_2_O_2_ solution for 15 min. After triple washing with distilled water, maize seeds were transferred to germinate in the dark on moist filter paper at 28°C. After 7 days, 18 germinated seeds from each of the 316 accessions were planted in a hydroponic chamber (69.5 × 43 × 20 cm) with an oxygen pump. The chamber contained 40L Hoagland's nutrient solution renewed every 2 days and was placed in a greenhouse with a long day (LD) light period (16/8 h) located at the Biotechnology Research Institute, Chinese Academy of Agricultural Sciences, Beijing, in 2019 and 2020. The EMS mutant *fut5* (EMS4‐270411) was sourced from the Maize EMS‐induced Mutant Database (http://maizeems.qlnu.edu.cn/) [74]. Subsequent sequencing (Table S7) confirmed the mutation. Homozygous mutants were purified through two generations of backcrossing and propagated in Hebei and Hainan provinces, China. Homozygous mutant *fut5* and B73 (wild type) seeds were germinated and grown in a hydroponic chamber. After 3 weeks, seedlings were harvested for scanner‐based image acquisition. Additionally, seedling samples were flash‐frozen in liquid nitrogen and stored at −80°C for subsequent RNA isolation.

### Determination of cellulose content

Cellulose content was measured using the Solarbio kit (BC4285) protocol. The samples were first dried at 80°C until a constant weight was reached, then crushed to obtain approximately 300 mg of material. Extraction solution 1 was added to the samples, which were then incubated in a water bath at 90°C for 20 min. After thermal treatment, the samples were centrifuged at 6000 × *g* for 10 min at room temperature, and the supernatant was discarded to collect the precipitate. The precipitate was washed twice with 1.5 mL of extraction solution 1. The cleaned precipitate was used as the crude cell wall fraction. The crude cell wall fraction was then soaked in 1 mL of extraction solution 2 for 15 h. After soaking, the samples were centrifuged again at 6000 × *g* for 10 min at room temperature. The supernatant was discarded, and the precipitate was washed twice with 0.5 mL of distilled water at 25°C. The precipitate was air‐dried to obtain the cell wall substance, which was then homogenized in 0.5 mL of distilled water using vortex mixing. After homogenization, 0.75 mL of concentrated sulfuric acid was added and mixed thoroughly. The supernatant was diluted 20‐fold with distilled water. The absorbance was measured at 620 nm.

### Determination of hemicellulose content

Hemicellulose content was measured using the protocol provided in the Solarbio kit (BC4440). Approximately 0.2 g of dried sample (dried at 80°C until constant weight) was crushed, treated with Reagent 1, and incubated in a 90°C water bath for 10 min. After centrifugation at 8000 × *g* for 10 min, the precipitate was collected, washed three times with 0.5 mL distilled water, and dried. Extraction solution 1 was added to the dried sample, followed by vortex mixing and incubation in a 90°C water bath for 1 h. After cooling to room temperature, extraction solution 2 was added, vortexed, and centrifuged at 8000 × *g* for 10 min. The supernatant was collected, mixed with reagent 2 and distilled water, and the absorbance was measured at 540 nm to determine hemicellulose content.

### Phenotyping and statistical analyses of maize heat tolerance

For the germination assay, seeds were sterilized with 10% H_2_O_2_ for 30 min and then rinsed three times with deionized water. The seeds were first germinated on moist filter paper at 25°C for 3‐4 days. Seedlings with uniform growth were then transferred to a 25°C incubator for hydroponic cultivation, under a light/dark cycle of 16/8 h. At the V3 stage, maize seedlings were moved to a 42°C incubator while maintaining the same light/dark cycle for two additional days. After phenotypic observation, leaves were photographed, and the total length and damaged length of the three blades were measured using ImageJ software [75].

### Phenotyping platform

At the V3 stage, seedlings were harvested, and roots were separated from shoots, then scanned using the HP700 scanner from the WinRHIZO system (2004b, Canada) to generate high‐resolution images. Three root types (primary, crown, lateral), each with three zones (Maturation, Elongation, Meristematic), were selected and preserved in FAA solution (5 mL 38% Formaldehyde, 5 mL Glacial Acetic Acid, 90 mL 70% Ethyl Alcohol, 5 mL Glycerinum). Weights of fresh shoots and roots were measured using an electronic balance and then dried at 60°C until a constant weight was achieved. The dried weights were also measured. For slice images, samples were embedded in 3% Agarose gel and cut with a Vibratome (Leica VT1200s) at a 50 μm step size. The entire slice was placed on a microscope slide, covered with a glass slide, and used for microscopic examination and image acquisition with a Fluorescence microscope (Leica DM2500).

### Image analysis and trait extraction

Root traits (R‐traits) were measured using root images from a scanner with the WinRHIZO system, encompassing total roots and primary roots. Weight traits (W‐traits) for roots and shoots were measured by an electronic balance, resulting in 16 R‐traits and 7 weight traits, respectively. Slice traits (S‐traits) were obtained from slice images using Image J software (https://imagej.net/software/imagej/), encompassing area‐related (e.g., root surface area), length‐related (e.g., Cortex diameter), and number‐related (e.g., xylem number) traits, totaling 108 S‐traits. In total, 131 traits (Table S2) were acquired for this study and utilized in a subsequent GWAS. Pearson correlation coefficients for R‐traits, W‐traits, and S‐traits were computed in R using the Hmisc package, available at https://github.com/harrelfe/Hmisc/.

### GWAS

Genotype data (2.65 M) from the association panel were obtained via the MaizeGO website (http://www.maizego.org/Resources.html). After filtering with a minor allelic frequency cutoff of 5%, 1.25 M variant maps were utilized. GWAS employed a mixed linear model accounting for population structure (*Q*) and relative kinship (*K*) in TASSEL software (v5.0) [76]. The effective numbers of independent SNPs (1,253,814) were estimated using the GEC tool [77] with the significant p‐value threshold set as 7.987 × 10^−7^ (Bonferroni correction of 1/*n*, where n represents independent markers). The decay distance of LD was calculated in the previous study [28], with significant SNPs within 50 kb binned into a QTL, designating the most significant SNP as the lead SNP. Haplotypes of candidate genes were determined from genotypes, and group comparisons of maize accessions with different haplotypes utilized the Wilcoxon rank‐sum test in the R package.

### Gene expression and quantification

RNA from *fut5* and B73 seedlings (each sample with three biological replicates) was extracted using the RNeasy Plus Mini Kit (Vazyme Biotech). High‐quality RNA‐generated libraries via the NEBNext Ultra RNA Library Prep Kit (NEB). Quantified libraries were sequenced on the Illumina HiSeq X‐ten sequencing platform. After filtering low‐quality (*Q* < 20) and adapter sequences, clean data were aligned to the B73 maize reference genome (V4) using HISAT2 software [78]. FeatureCounts tallied read numbers mapped to each gene, normalized with fragments per kilobase of exon model per million mapped reads (FPKM) [79]. Pearson correlation coefficient between samples was calculated in R software and visualized using the pheatmap package.

### Differential expression and gene ontology (GO) enrichment analysis

Differential expression genes were identified with a fold change cutoff 2 and statistical significance *p* < 0.05 using DESeq.2 software [80]. GO enrichment analysis was performed using an online web server AgriGO *v2* (http://systemsbiology.cau.edu.cn/ agriGOv2/) with default parameters.

### Haplotype analysis of the *FUT5* gene and association with traits in maize

Based on 10 non‐synonymous SNPs, the 316 accessions were categorized into eight haplotypes, and root traits (R‐traits) were compared among these haplotypes. A haplotype network was constructed using the PopART software (https://popart.maths.otago.ac.nz/ download/) employing the integer NJ Net method.

### Detection of selection signals of *FUT5* gene

Genomic sequences from 316 maize accessions underwent genetic diversity analysis. Nucleotide diversity (π) and Tajima's D in the 2M region encompassing fut5 were computed for the entire population, tropical/subtropical, and temperate subspecies. Calculations utilized a 50 kb sliding window and 50 kb step, performed with VCFtools (https://github.com/ vcftools/vcftools).

### Prediction of maize root architecture using slice‐traits based on machine learning

Utilizing the bagging tree machine learning algorithm, a model suitable for complex data processing and prediction [81] was constructed. The algorithm was implemented using the caret package in R (https://CRAN.R-project.org/ package = caret). The raw data of maize accessions were subsequently scaled. The bagging tree machine learning algorithm predicted the 16 root traits (R‐traits) based on 108 slice traits (S‐traits). The model's construction and validation employed a 10‐fold cross‐validation strategy. The average Pearson correlation coefficient of 10 models assessed the model's performance. All plots were generated using the ggplot2 package in R.

## AUTHOR CONTRIBUTIONS

**Weijun Guo**: Software; data curation; investigation; visualization; methodology. **Fanhua Wang**: Validation; investigation; visualization. **Jianyue Lv**: Visualization. **Jia Yu**: Data curation; Validation; formal analysis. **Yue Wu**: Project administration. **Hada Wuriyanghan**: Data curation; investigation; validation; methodology. **Liang Le**: Conceptualization; writing—original draft; writing—review and editing. **Li Pu**: Conceptualization; funding acquisition; writing—review and editing; writing—original draft; resources; supervision.

## CONFLICT OF INTEREST STATEMENT

The authors declare no conflicts of interest.

## ETHICS STATEMENT

No animals or humans were involved in this study.

## Supporting information


**Figure S1.** Comparison of 16 R‐traits between tropical/subtropical and temperate maize inbred lines.
**Figure S2.** Comparison of 7 W‐traits between tropical/subtropical and temperate maize inbred lines.
**Figure S3.** Comparison of 36 S‐traits in primary roots between tropical/subtropical and temperate maize inbred lines.
**Figure S4.** Comparison of 36 S‐traits in lateral roots between tropical/subtropical and temperate maize inbred lines.
**Figure S5.** Comparison of 36 S‐traits in crown roots between tropical/subtropical and temperate maize inbred lines.
**Figure S6.** The relevance of the R‐traits and S‐traits in primary roots.
**Figure S7.** GWAS identification of candidate genes for variation in W‐traits and R‐traits, in maize.
**Figure S8.** Comparisons of candidate genes with other GWAS results in maize roots.
**Figure S9.** Local Manhattan plot and LD statistic *r* ^2^ values (left) for the *ereb160* gene associated with a W‐trait, weight of fresh roots.

**Table S1.** The information of 316 maize accessions in the panel.
**Table S2.** A list of R‐traits, W‐traits, and S‐traits was analyzed by WinRhizo and their definitions.
**Table S3.** Root traits with means of all inbred lines, minimums, maximums, variance, and coefficient of variation (CV).
**Table S4.** Pearson correlation coefficient among R‐traits, W‐traits, and plant architecture‐related traits.
**Table S5.** Summary of trait associated candidate genes.
**Table S6.** Candidate genes associated with all traits.
**Table S7.** Primer sequences used in the present study.

## Data Availability

All the images, phenotypic data, and RNAs‐seq data are available at https://doi.org/10.6084/m9.figshare.27605208.v1. The original data and code for GWAS analysis pipelines can be downloaded at https://github.com/GUOWEIJUN/maizerootphenomics. Supplementary materials (figures, tables, graphical abstract, slides, videos, Chinese translated version, and update materials) may be found in the online DOI or iMeta Science http://www.imeta.science/. The data that support the findings of this study are openly available on Github at https://github.com/GUOWEIJUN/maizerootphenomics.

## References

[1] Lynch, Jonathan P. , and Kathleen M. Brown . 2012. “New Roots for Agriculture: Exploiting the Root Phenome.” Philosophical Transactions of the Royal Society B: Biological Sciences 367: 1598–1604. 10.1098/rstb.2011.0243 PMC3321693 22527403

[2] Liu, Suxing , Carlos Sherard Barrow , Meredith Hanlon , Jonathan P. Lynch , and Alexander Bucksch . 2021. “DIRT/3D: 3D Root Phenotyping for Field‐Grown Maize (*Zea Mays*).” Plant Physiology 187: 739–757. 10.1093/plphys/kiab311 34608967 PMC8491025

[3] Ren, Wei , Longfei Zhao , Jiaxing Liang , Lifeng Wang , Limei Chen , Pengcheng Li , Zhigang Liu , et al. 2022. “Genome‐Wide Dissection of Changes in Maize Root System Architecture During Modern Breeding.” Nature Plants 8: 1408–1422. 10.1038/s41477-022-01274-z 36396706

[4] Zheng, Zihao , Bufei Guo , Somak Dutta , Vivekananda Roy , Huyu Liu , and Patrick S. Schnable . 2023. “The 2020 Derecho Revealed Limited Overlap Between Maize Genes Associated With Root Lodging and Root System Architecture.” Plant Physiology 192: 2394–2403. 10.1093/plphys/kiad194 36974884 PMC10315264

[5] Lynch, Jonathan P . 2019. “Root Phenotypes for Improved Nutrient Capture: An Underexploited Opportunity for Global Agriculture.” New Phytologist 223: 548–564. 10.1111/nph.15738 30746704

[6] Wu, Qian , Jie Wu , Pengcheng Hu , Weixin Zhang , Yuntao Ma , Kun Yu , Yan Guo , et al. 2023. “Quantification of the Three‐Dimensional Root System Architecture Using an Automated Rotating Imaging System.” Plant Methods 19: 11. 10.1186/s13007-023-00988-1 36732764 PMC9896698

[7] Bucksch, A. , J. Burridge , L. M. York , A. Das , E. Nord , J. S. Weitz , and J. P. Lynch . 2014. “Image‐Based High‐Throughput Field Phenotyping of Crop Roots.” Plant Physiology 166: 470–486. 10.1104/pp.114.243519 25187526 PMC4213080

[8] Bucksch, Alexander , Abhiram Das , Hannah Schneider , Nirav Merchant , and Joshua S. Weitz . 2017. “Overcoming the Law of the Hidden in Cyberinfrastructures.” Trends in Plant Science 22: 117–123. 10.1016/j.tplants.2016.11.014 28027865

[9] Le Marié, Chantal , Norbert Kirchgessner , Daniela Marschall , Achim Walter , and Andreas Hund . 2014. “Rhizoslides: Paper‐Based Growth System for Non‐Destructive, High Throughput Phenotyping of Root Development by Means of Image Analysis.” Plant Methods 10: 13. 10.1186/1746-4811-10-13 25093035 PMC4105838

[10] Mathieu, Laura , Guillaume Lobet , Pierre Tocquin , and Claire Périlleux . 2015. “ ‘Rhizoponics’: A Novel Hydroponic Rhizotron for Root System Analyses on Mature *Arabidopsis Thaliana* Plants.” Plant Methods 11: 3. 10.1186/s13007-015-0046-x 25657812 PMC4318444

[11] Galkovskyi, Taras , Yuriy Mileyko , Alexander Bucksch , Brad Moore , Olga Symonova , Charles A. Price , Christopher N. Topp , et al. 2012. “GiA Roots: Software for the High Throughput Analysis of Plant Root System Architecture.” BMC Plant Biology 12: 116. 10.1186/1471-2229-12-116 22834569 PMC3444351

[12] Clark, Randy T. , Robert B. Maccurdy , Janelle K. Jung , Jon E. Shaff , Susan R. Mccouch , Daniel J. Aneshansley , and Leon V. Kochian . 2011. “Three‐Dimensional Root Phenotyping With a Novel Imaging and Software Platform.” Plant Physiology 156: 455–465. 10.1104/pp.110.169102 21454799 PMC3177249

[13] Fukatsu, Tokihiro , Tomonari Watanabe , Haoming Hu , Hideo Yoichi , and Masayuki Hirafuji . 2012. “Field Monitoring Support System for the Occurrence of *Leptocorisa chinensis* Dallas (Hemiptera: Alydidae) Using Synthetic Attractants, Field Servers, and Image Analysis.” Computers and Electronics in Agriculture 80: 8–16. 10.1016/j.compag.2011.10.005

[14] Zhang, Xiaomin , Yue Mi , Hude Mao , Shengxue Liu , Limei Chen , and Feng Qin . 2020. “Genetic Variation in ZmTIP1 Contributes to Root Hair Elongation and Drought Tolerance in Maize.” Plant Biotechnology Journal 18: 1271–1283. 10.1111/pbi.13290 31692165 PMC7152618

[15] Schneider, Hannah M. , Vai Sa Nee Lor , Meredith T. Hanlon , Alden Perkins , Shawn M. Kaeppler , Aditi N. Borkar , Rahul Bhosale , et al. 2022. “Root Angle in Maize Influences Nitrogen Capture and Is Regulated by Calcineurin B‐Like Protein(CBL)‐Interacting Serine/Threonine‐Protein Kinase 15 (ZmCIPK15).” Plant, Cell & Environment 45: 837–853. 10.1111/pce.14135 PMC9544310 34169548

[16] Pace, Jordon , Nigel Lee , Hsiang Sing Naik , Baskar Ganapathysubramanian , and Thomas Lübberstedt . 2014. “Analysis of Maize (*Zea Mays L*.) Seedling Roots With the High‐Throughput Image Analysis Tool ARIA (Automatic Root Image Analysis).” PLoS One 9: e108255. 10.1371/journal.pone.0108255 25251072 PMC4176968

[17] Pace, Jordon , Candice Gardner , Cinta Romay , Baskar Ganapathysubramanian , and Thomas Lübberstedt . 2015. “Genome‐Wide Association Analysis of Seedling Root Development in Maize (*Zea Mays L*.).” BMC Genomics 16: 47. 10.1186/s12864-015-1226-9 25652714 PMC4326187

[18] Hochholdinger, Frank . 2009. “The Maize Root System: Morphology, Anatomy, and Genetics.” In Handbook of Maize: Its Biology, edited by J. L. Bennetzen and S. C. Hake , 145–160. Springer New York. 10.1007/978-0-387-79418-1_8

[19] Wang, Ting , Fanhua Wang , Shuhan Deng , Kailai Wang , Dan Feng , Fan Xu , Weijun Guo , et al. 2025. “Single‐Cell Transcriptomes Reveal Spatiotemporal Heat Stress Response in Maize Roots.” Nature Communications 16: 177. 10.1038/s41467-024-55485-3 PMC11697069 39747108

[20] Lynch, J . 1995. “Root Architecture and Plant Productivity.” Plant Physiology 109: 7–13. 10.1104/pp.109.1.7 12228579 PMC157559

[21] Evans, M. M. S. , and R. S. Poethig . 1995. “Gibberellins Promote Vegetative Phase‐Change and Reproductive Maturity in Maize.” Plant Physiology 108: 475–487. 10.1104/pp.108.2.475 7610158 PMC157366

[22] Suzuki, Masaharu , Yutaka Sato , Shan Wu , Byung‐Ho Kang , and Donald R. Mccarty . 2015. “Conserved Functions of the MATE Transporter BIG EMBRYO1 in Regulation of Lateral Organ Size and Initiation Rate.” Plant Cell 27: 2288–2300. 10.1105/tpc.15.00290 26276834 PMC4568504

[23] Han, Bin , Sheng Xu , Yan‐Jie Xie , Jing‐Jing Huang , Li‐Juan Wang , Zheng Yang , Chang‐He Zhang , et al. 2012. “ZmHO‐1, a Maize Haem oxygenase‐1 Gene, Plays a Role in Determining Lateral Root Development.” Plant Science 184: 63–74. 10.1016/j.plantsci.2011.12.012 22284711

[24] Yan, Jun , and Xiangfeng Wang . 2023. “Machine Learning Bridges Omics Sciences and Plant Breeding.” Trends in Plant Science 28: 199–210. 10.1016/j.tplants.2022.08.018 36153276

[25] Hickey, John M. , Tinashe Chiurugwi , Ian Mackay , and Wayne Powell , Implementing Genomic Selection in Cgiar Breeding Programs Workshop Participants . 2017. “Genomic Prediction Unifies Animal and Plant Breeding Programs to Form Platforms for Biological Discovery.” Nature Genetics 49: 1297–1303. 10.1038/ng.3920 28854179

[26] González‐Recio, Oscar , Guilherme J. M. Rosa , and Daniel Gianola . 2014. “Machine Learning Methods and Predictive Ability Metrics for Genome‐Wide Prediction of Complex Traits.” Livestock Science 166: 217–231. 10.1016/j.livsci.2014.05.036

[27] Desta, Zeratsion Abera , and Rodomiro Ortiz . 2014. “Genomic Selection: Genome‐Wide Prediction in Plant Improvement.” Trends in Plant Science 19: 592–601. 10.1016/j.tplants.2014.05.006 24970707

[28] Wang, Weixuan , Weijun Guo , Liang Le , Jia Yu , Yue Wu , Dongwei Li , Yifan Wang , et al. 2023. “Integration of High‐Throughput Phenotyping, GWAS, and Predictive Models Reveals the Genetic Architecture of Plant Height in Maize.” Molecular Plant 16: 354–373. 10.1016/j.molp.2022.11.016 36447436 PMC11801313

[29] Lynch, Jonathan P . 2013. “Steep, Cheap and Deep: An Ideotype to Optimize Water and N Acquisition by Maize Root Systems.” Annals of Botany 112: 347–357. 10.1093/aob/mcs293 23328767 PMC3698384

[30] Zhao, Jin , Qing‐Wu Xue , Kirk E. Jessup , Xiao‐Bo Hou , Bao‐Zhen Hao , Thomas H. Marek , Wen‐Wei Xu , et al. 2018. “Shoot and Root Traits in Drought Tolerant Maize (*Zea mays L*.) Hybrids.” Journal of Integrative Agriculture 17: 1093–1105. 10.1016/S2095-3119(17)61869-0

[31] Nass, H. G. , and M. S. Zuber . 1971. “Correlation of Corn (*Zea Mays L*.) Roots Early in Development to Mature Root Development^1^ .” Crop Science 11: 655–658. 10.2135/cropsci1971.0011183X001100050015x

[32] Yang, Ning , Yanli Lu , Xiaohong Yang , Juan Huang , Yang Zhou , Farhan Ali , Weiwei Wen , et al. 2014. “Genome Wide Association Studies Using a New Nonparametric Model Reveal the Genetic Architecture of 17 Agronomic Traits in an Enlarged Maize Association Panel.” Plos Genetics 10: e1004573. 10.1371/journal.pgen.1004573 25211220 PMC4161304

[33] He, Kunhui , Zheng Zhao , Wei Ren , Zhe Chen , Limei Chen , Fanjun Chen , Guohua Mi , Qingchun Pan , and Lixing Yuan . 2023. “Mining Genes Regulating Root System Architecture in Maize Based on Data Integration Analysis.” Theoretical and Applied Genetics 136: 127. 10.1007/s00122-023-04376-0 37188973

[34] Yu, Feng , Kun Liang , Tian Fang , Hailiang Zhao , Xuesong Han , Manjun Cai , and Fazhan Qiu . 2019. “A Group VII Ethylene Response Factor Gene, ZmEREB180, Coordinates Waterlogging Tolerance in Maize Seedlings.” Plant Biotechnology Journal 17: 2286–2298. 10.1111/pbi.13140 31033158 PMC6835127

[35] Li, Li , Stefan Hey , Sanzhen Liu , Qiang Liu , Colton Mcninch , Heng‐Cheng Hu , and Tsui‐Jung Wen , et al. 2016. “Characterization of Maize roothairless6 Which Encodes a D‐Type Cellulose Synthase and Controls the Switch From Bulge Formation to Tip Growth.” Scientific Reports 6: 34395. 10.1038/srep34395 27708345 PMC5052636

[36] Evans, Matthew M. S. , and R. Scott Poethig . 1997. “The viviparous8 Mutation Delays Vegetative Phase Change and Accelerates the Rate of Seedling Growth in Maize.” The Plant Journal 12: 769–779. 10.1046/j.1365-313X.1997.12040769.x

[37] Nestler, Josefine , Sanzhen Liu , Tsui‐Jung Wen , Anja Paschold , Caroline Marcon , Ho Man Tang , Delin Li , et al. 2014. “Roothairless5, Which Functions in Maize (*Zea mays* L.) Root Hair Initiation and Elongation Encodes a Monocot‐Specific NADPH Oxidase.” The Plant Journal 79: 729–740. 10.1111/tpj.12578 24902980

[38] Li, Zhaoxia , Xinrui Zhang , Yajie Zhao , Yujie Li , Guangfeng Zhang , Zhenghua Peng , and Juren Zhang . 2018. “Enhancing Auxin Accumulation in Maize Root Tips Improves Root Growth and Dwarfs Plant Height.” Plant Biotechnology Journal 16: 86–99. 10.1111/pbi.12751 28499064 PMC5785362

[39] Stephenson, Elizabeth , Stacey Estrada , Xin Meng , Jesse Ourada , Michael G. Muszynski , Jeffrey E. Habben , and Olga N. Danilevskaya . 2019. “Over‐Expression of the Photoperiod Response Regulator ZmCCT10 Modifies Plant Architecture, Flowering Time and Inflorescence Morphology in Maize.” PLoS One 14: e0203728. 10.1371/journal.pone.0203728 30726207 PMC6364868

[40] Chen, Zhe , Junli Sun , Dongdong Li , Pengcheng Li , Kunhui He , Farhan Ali , Guohua Mi , et al. 2022. “Plasticity of Root Anatomy during Domestication of a Maize‐Teosinte Derived Population.” Journal of Experimental Botany 73: 139–153. 10.1093/jxb/erab406 34487165

[41] Moussa, Abdourazak Alio , Ajmal Mandozai , Yukun Jin , Jing Qu , Qi Zhang , He Zhao , Gulaqa Anwari , et al. 2021. “Genome‐Wide Association Screening and Verification of Potential Genes Associated With Root Architectural Traits in Maize (*Zea mays L*.) at Multiple Seedling Stages.” BMC Genomics 22: 558. 10.1186/s12864-021-07874-x 34284723 PMC8290564

[42] Cao, Liru , Feiyu Ye , Abbas Muhammad Fahim , Chenchen Ma , Yunyun Pang , Xin Zhang , Qianjin Zhang , and Xiaomin Lu . 2024. “Transcription Factor ZmDof22 Enhances Drought Tolerance by Regulating Stomatal Movement and Antioxidant Enzymes Activities in Maize (*Zea mays L*.).” Theoretical and Applied Genetics 137: 132. 10.1007/s00122-024-04625-w 38750241

[43] Luo, Bowen , Ziqi Zhang , Binyang Li , Haiying Zhang , Junchi Ma , Jing Li , Zheng Han , et al. 2023. “Chromatin Remodeling Analysis Reveals the RdDM Pathway Responds to Low‐Phosphorus Stress in Maize.” The Plant Journal 117: 33–52. 10.1111/tpj.16468 37731059

[44] Lee, Dong‐Keun , Harin Jung , Geupil Jang , Jin Seo Jeong , Youn Shic Kim , Sun‐Hwa Ha , Yang Do Choi , and Ju‐Kon Kim . 2016. “Overexpression of the OsERF71 Transcription Factor Alters Rice Root Structure and Drought Resistance.” Plant Physiology 172: 575–588. 10.1104/pp.16.00379 27382137 PMC5074616

[45] Gevers, Helmut O . 1975. “A New Major Gene for Resistance to Helminthosporium Turcicum Leaf Blight of Maize.” Plant Disease Reporter 59: 296–299.

[46] Hurni, Severine , Daniela Scheuermann , Simon G. Krattinger , Bettina Kessel , Thomas Wicker , Gerhard Herren , Mirjam N. Fitze , et al. 2015. “The Maize Disease Resistance Gene Htn1 Against Northern Corn Leaf Blight Encodes a Wall‐Associated Receptor‐Like Kinase.” Proceedings of the National Academy of Sciences 112: 8780–8785. 10.1073/pnas.1502522112 PMC4507197 26124097

[47] Sarria, Rodrigo , Tanya A. Wagner , Malcolm A. O'Neill , Ahmed Faik , Curtis G. Wilkerson , Kenneth Keegstra , and Natasha V. Raikhel . 2001. “Characterization of a Family of Arabidopsis Genes Related to Xyloglucan fucosyltransferase1.” Plant Physiology 127: 1595–1606. 10.1104/pp.010596 11743104 PMC133564

[48] Soto, Maria J. , Pradeep Kumar Prabhakar , Hsin‐Tzu Wang , Jason Backe , Digantkumar Chapla , Max Bartetzko , Ian M. Black , et al. 2021. “AtFUT4 and AtFUT6 Are Arabinofuranose‐Specific Fucosyltransferases.” Frontiers in Plant Science 12: 589518. 10.3389/fpls.2021.589518 33633757 PMC7900004

[49] Wang, Huai , Anthony J. Studer , Qiong Zhao , Robert Meeley , and John F. Doebley . 2015. “Evidence That the Origin of Naked Kernels During Maize Domestication Was Caused by a Single Amino Acid Substitution in tga1.” Genetics 200: 965–974. 10.1534/genetics.115.175752 25943393 PMC4512555

[50] Ma, Haizhen , Can Liu , Zhaoxia Li , Qijun Ran , Guangning Xie , Baomei Wang , Shuang Fang , Jinfang Chu , and Juren Zhang . 2018. “ZmbZIP4 Contributes to Stress Resistance in Maize by Regulating ABA Synthesis and Root Development.” Plant Physiology 178: 753–770. 10.1104/pp.18.00436 30126870 PMC6181033

[51] Mori, Masaki , Takahito Nomura , Hisako Ooka , Masumi Ishizaka , Takao Yokota , Kazuhiko Sugimoto , Ken Okabe , et al. 2002. “Isolation and Characterization of a Rice Dwarf Mutant With a Defect in Brassinosteroid Biosynthesis.” Plant Physiology 130: 1152–1161. 10.1104/pp.007179 12427982 PMC166636

[52] Liu, Ying , Zhongtao Jia , Xuelian Li , Zhangkui Wang , Fanjun Chen , Guohua Mi , Brian Forde , Hideki Takahashi , and Lixing Yuan . 2020. “Involvement of a Truncated MADS‐box Transcription Factor ZmTMM1 in Root Nitrate Foraging.” Journal of Experimental Botany 71: 4547–4561. 10.1093/jxb/eraa116 32133500 PMC7382388

[53] Xu, Changzheng , Huanhuan Tai , Muhammad Saleem , Yvonne Ludwig , Christine Majer , Kenneth W. Berendzen , Kerstin A. Nagel , et al. 2015. “Cooperative Action of the Paralogous Maize Lateral Organ Boundaries (LOB) Domain Proteins RTCS and RTCL in Shoot‐Borne Root Formation.” New Phytologist 207: 1123–1133. 10.1111/nph.13420 25902765

[54] Perrin, Robyn M. , Amy E. Derocher , Maor Bar‐Peled , Weiqing Zeng , Lorena Norambuena , Ariel Orellana , Natasha V. Raikhel , and Kenneth Keegstra . 1999. “Xyloglucan Fucosyltransferase, an Enzyme Involved in Plant Cell Wall Biosynthesis.” Science 284: 1976–1979. 10.1126/science.284.5422.1976 10373113

[55] Ma, Chuang , Hao Helen Zhang , and Xiangfeng Wang . 2014. “Machine Learning for Big Data Analytics in Plants.” Trends in Plant Science 19: 798–808. 10.1016/j.tplants.2014.08.004 25223304

[56] Hiddar, Houda , Sajid Rehman , Berhane Lakew , Ramesh Pal Singh Verma , Muamar Al‐Jaboobi , Adil Moulakat , Zakaria Kehel , et al. 2021. “Assessment and Modeling Using Machine Learning of Resistance to Scald (Rhynchosporium commune) in Two Specific Barley Genetic Resources Subsets.” Scientific Reports 11: 15967. 10.1038/s41598-021-94587-6 34354105 PMC8342473

[57] Ghimire, Bardan , John Rogan , Víctor Rodríguez Galiano , Prajjwal Panday , and Neeti Neeti . 2012. “An Evaluation of Bagging, Boosting, and Random Forests for Land‐Cover Classification in Cape Cod, Massachusetts, USA.” Giscience & Remote Sensing 49: 623–643. 10.2747/1548-1603.49.5.623

[58] Zhu, Jinming , Paul A. Ingram , Philip N. Benfey , and Tedd Elich . 2011. “From Lab to Field, New Approaches to Phenotyping Root System Architecture.” Current Opinion in Plant Biology 14: 310–317. 10.1016/j.pbi.2011.03.020 21530367

[59] Ma, Langlang , Chunyan Qing , Ursula Frei , Yaou Shen , and Thomas Lübberstedt . 2020. “Association Mapping for Root System Architecture Traits Under Two Nitrogen Conditions in Germplasm Enhancement of Maize Doubled Haploid Lines.” The Crop Journal 8: 213–226. 10.1016/j.cj.2019.11.004

[60] Wang, Xianglan , Hongwei Wang , Shengxue Liu , Ali Ferjani , Jiansheng Li , Jianbing Yan , Xiaohong Yang , and Feng Qin . 2016. “Genetic Variation in ZmVPP1 Contributes to Drought Tolerance in Maize Seedlings.” Nature Genetics 48: 1233–1241. 10.1038/ng.3636 27526320

[61] Sun, Daqiu , Sibo Chen , Zhenhai Cui , Jingwei Lin , Meiling Liu , Yueting Jin , Ao Zhang , et al. 2022. “Genome‐Wide Association Study Reveals the Genetic Basis of Brace Root Angle and Diameter in Maize.” Frontiers in Genetics 13: 963852. 10.3389/fgene.2022.963852 36276979 PMC9582141

[62] Sun, Guangchao , Huihui Yu , Peng Wang , Martha Lopez‐Guerrero , Ravi V. Mural , Olivier N. Mizero , Marcin Grzybowski , et al. 2023. “A Role for Heritable Transcriptomic Variation in Maize Adaptation to Temperate Environments.” Genome Biology 24: 55. 10.1186/s13059-023-02891-3 36964601 PMC10037803

[63] Liu, Lin , Lu‐Guang Jiang , Jin‐Hong Luo , Ai‐Ai Xia , Li‐Qun Chen , and Yan He . 2021. “Genome‐Wide Association Study Reveals the Genetic Architecture of Root Hair Length in Maize.” BMC Genomics 22: 664. 10.1186/s12864-021-07961-z 34521344 PMC8442424

[64] Maurel, Christophe , and Philippe Nacry . 2020. “Root Architecture and Hydraulics Converge for Acclimation to Changing Water Availability.” Nature Plants 6: 744–749. 10.1038/s41477-020-0684-5 32601421

[65] Hund, Andreas , Regina Reimer , and Rainer Messmer . 2011. “A Consensus Map of QTLs Controlling the Root Length of Maize.” Plant and Soil 344: 143–158. 10.1007/s11104-011-0735-9

[66] Hochholdinger, Frank , Peng Yu , and Caroline Marcon . 2018. “Genetic Control of Root System Development in Maize.” Trends in Plant Science 23: 79–88. 10.1016/j.tplants.2017.10.004 29170008

[67] Bray, Adam L. , and Christopher N. Topp . 2018. “The Quantitative Genetic Control of Root Architecture in Maize.” Plant and Cell Physiology 59: 1919–1930. 10.1093/pcp/pcy141 30020530 PMC6178961

[68] Qin, Hua , Juan Wang , Xinbing Chen , Fangfang Wang , Peng Peng , Yun Zhou , Yuchen Miao , et al. 2019. “Rice OsDOF15 Contributes to Ethylene‐Inhibited Primary Root Elongation Under Salt Stress.” New Phytologist 223: 798–813. 10.1111/nph.15824 30924949

[69] Li, Qing , Steven R. Eichten , Peter J. Hermanson , Virginia M. Zaunbrecher , Jawon Song , Jennifer Wendt , Heidi Rosenbaum , et al. 2014. “Genetic Perturbation of the Maize Methylome.” Plant Cell 26: 4602–4616. 10.1105/tpc.114.133140 25527708 PMC4311211

[70] Long, Jin Cheng , Ai Ai Xia , Jing Han Liu , Ju Li Jing , Ya Zhong Wang , Chuang Ye Qi , and Yan He . 2019. “Decrease in DNA Methylation 1 (DDM1) Is Required for the Formation of mCHH Islands in Maize.” Journal of Integrative Plant Biology 61: 749–764. 10.1111/jipb.12733 30387549

[71] Kozlova, Liudmila V. , Alsu R. Nazipova , Oleg V. Gorshkov , Anna A. Petrova , and Tatyana A. Gorshkova . 2020. “Elongating Maize Root: Zone‐Specific Combinations of Polysaccharides From Type I and Type II Primary Cell Walls.” Scientific Reports 10: 10956. 10.1038/s41598-020-67782-0 32616810 PMC7331734

[72] Pang, Shengyan , Hongyan Zheng , Jiankui Zhang , Xiaotian Ren , Xuefeng Zong , Junjie Zou , and Lei Wang . 2024. “Function Analysis of a Maize Endo‐1,4‐β‐xylanase Gene ZmHSL in Response to High‐Temperature Stress.” International Journal of Molecular Sciences 25: 8834. 10.3390/ijms25168834 39201520 PMC11354693

[73] Li, Ze , Zerui Li , Yulong Ji , Chunyu Wang , Shufang Wang , Yiting Shi , Jie Le , and Mei Zhang . 2024. “The Heat Shock Factor 20‐HSF4‐Cellulose Synthase A2 Module Regulates Heat Stress Tolerance in Maize.” Plant Cell 36: 2652–2667. 10.1093/plcell/koae106 38573521 PMC11218781

[74] Lu, Xiaoduo , Jisheng Liu , Wen Ren , Qun Yang , Zhenguang Chai , Rumei Chen , Lei Wang , et al. 2018. “Gene‐Indexed Mutations in Maize.” Molecular Plant 11: 496–504. 10.1016/j.molp.2017.11.013 29223623

[75] Jiang, Haifang , Yiting Shi , Jingyan Liu , Zhen Li , Diyi Fu , Shifeng Wu , Minze Li , et al. 2022. “Natural Polymorphism of ZmICE1 Contributes to Amino Acid Metabolism That Impacts Cold Tolerance in Maize.” Nature Plants 8: 1176–1190. 10.1038/s41477-022-01254-3 36241735

[76] Bradbury, Peter J. , Zhiwu Zhang , Dallas E. Kroon , Terry M. Casstevens , Yogesh Ramdoss , and Edward S. Buckler . 2007. “TASSEL: Software for Association Mapping of Complex Traits in Diverse Samples.” Bioinformatics 23: 2633–2635. 10.1093/bioinformatics/btm308 17586829

[77] Li, Miao‐Xin , Juilian M. Y. Yeung , Stacey S. Cherny , and Pak C. Sham . 2012. “Evaluating the Effective Numbers of Independent Tests and Significant *P*‐Value Thresholds in Commercial Genotyping Arrays and Public Imputation Reference Datasets.” Human Genetics 131: 747–756. 10.1007/s00439-011-1118-2 22143225 PMC3325408

[78] Kim, Daehwan , Joseph M. Paggi , Chanhee Park , Christopher Bennett , and Steven L. Salzberg . 2019. “Graph‐Based Genome Alignment and Genotyping With HISAT2 and HISAT‐Genotype.” Nature Biotechnology 37: 907–915. 10.1038/s41587-019-0201-4 PMC7605509 31375807

[79] Liao, Yang , Gordon K. Smyth , and Wei Shi . 2014. “Featurecounts: An Efficient General Purpose Program for Assigning Sequence Reads to Genomic Features.” Bioinformatics 30: 923–930. 10.1093/bioinformatics/btt656 24227677

[80] Love, Michael I. , Wolfgang Huber , and Simon Anders . 2014. “Moderated Estimation of Fold Change and Dispersion for RNA‐seq Data With DESeq. 2.” Genome Biology 15: 550. 10.1186/s13059-014-0550-8 25516281 PMC4302049

[81] Yaman, Emine , and Abdulhamit Subasi . 2019. “Comparison of Bagging and Boosting Ensemble Machine Learning Methods for Automated EMG Signal Classification.” Biomed Research International 2019: 9152506. 10.1155/2019/9152506 31828145 PMC6885261

