# Supplementary material for: Phenotyping, genome‐wide dissection, and prediction of maize root architecture for temperate adaptability
Source: Imeta. 2025 Mar 13;4(2):e70015. doi: 10.1002/imt2.70015 (PMC11995184; doi:10.1002/imt2.70015)
Supplement: Supplementary file 1 — Figure S1. Comparison of 16 R‐traits between tropical/subtropical and temperate maize inbred lines. Figure S2. Comparison of 7 W‐traits between tropical/subtropical and temperate maize inbred lines. Figure S3. Comparison of 36 S‐traits in primary roots between tropical/subtropical and temperate maize inbred lines. Figure S4. Comparison of 36 S‐traits in lateral roots between tropical/subtropical and temperate maize inbred lines. Figure S5. Comparison of 36 S‐traits in crown roots between tropical/subtropical and temperate maize inbred lines. Figure S6. The relevance of the R‐traits and S‐traits in primary roots. Figure S7. GWAS identification of candidate genes for variation in W‐traits and R‐traits, in maize. Figure S8. Comparisons of candidate genes with other GWAS results in maize roots. Figure S9. Local Manhattan plot and LD statistic r 2 values (left) for the ereb160 gene associated with a W‐trait, weight of fresh roots. [file IMT2-4-e70015-s002.doc]

**Supporting information to**

**Phenotyping, Genome-wide Dissection, and Prediction of Maize Root Architecture for Temperate Adaptability**

Weijun Guo^1,2, 3^**^#^**, Fanhua Wang^1, 2^**^#^**, Jianyue Lv^1^**^#^**, Jia Yu^1^, Yue Wu^1^, Hada Wuriyanghan^2^, Liang Le^1*^, Li Pu^1*^

^1^Biotechnology Research Institute, Chinese Academy of Agricultural Sciences, Beijing, 100081, China

^2^School of Life Science, Inner Mongolia University, Hohhot 010021 China

^3^College of Life and Environmental Sciences, Hangzhou Normal University, Hangzhou 311121, China

**^#^**These authors contributed equally to this work

*Correspondence: [puli@caas.cn](mailto:puli@caas.cn) (Li Pu), [leliang@caas.cn](mailto:leliang@caas.cn) (Liang Le)


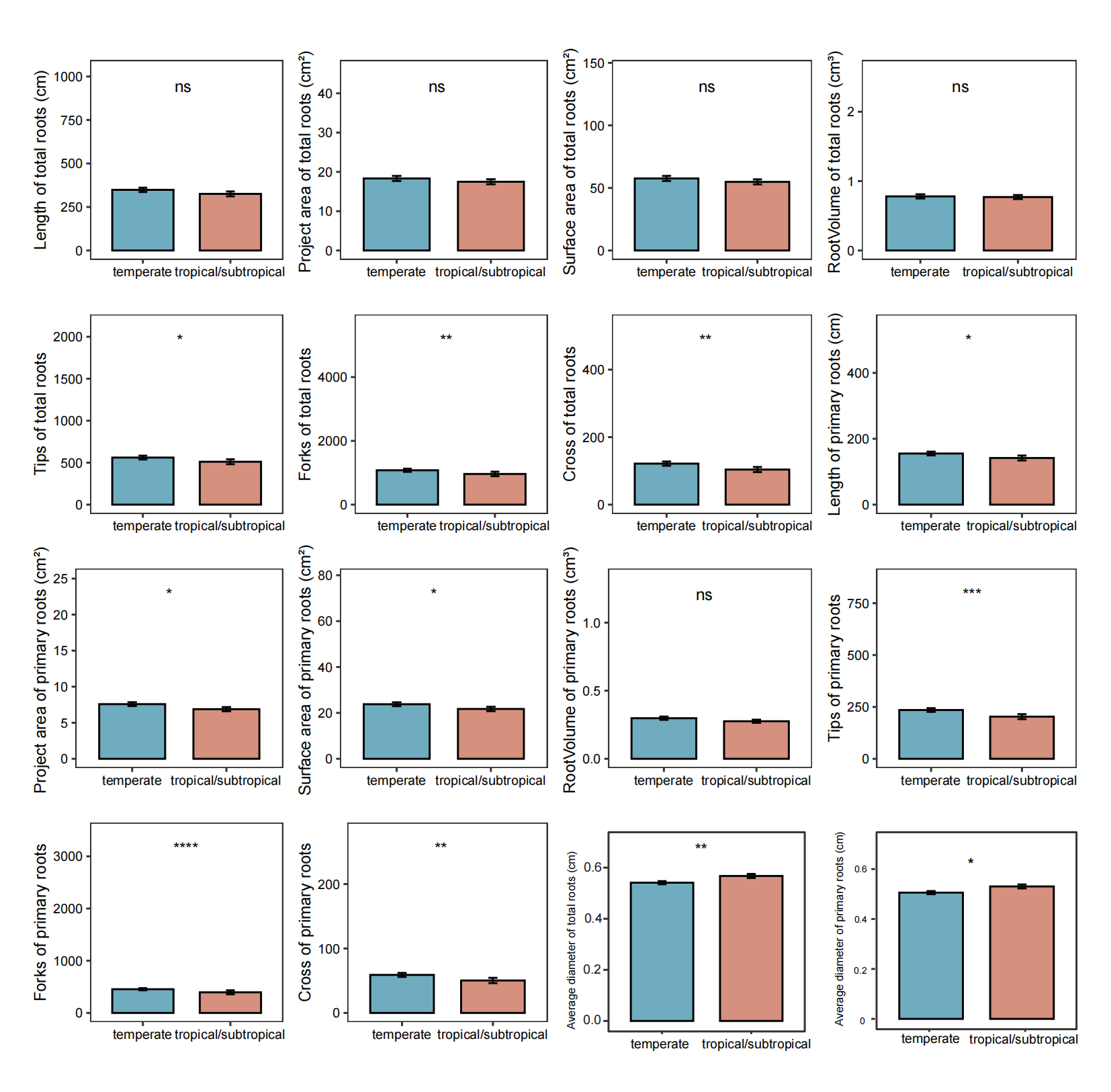


**Figure S1. Comparison of 16 R-traits between tropical/subtropical and temperate maize inbred lines.** The figure presents a series of violin plots comparing various root traits, including total root length, surface area, volume, and other parameters, between tropical/subtropical (in orange) and temperate (in blue) maize inbred lines. Statistical significance for each trait is indicated by the *p*-value from the T-test.


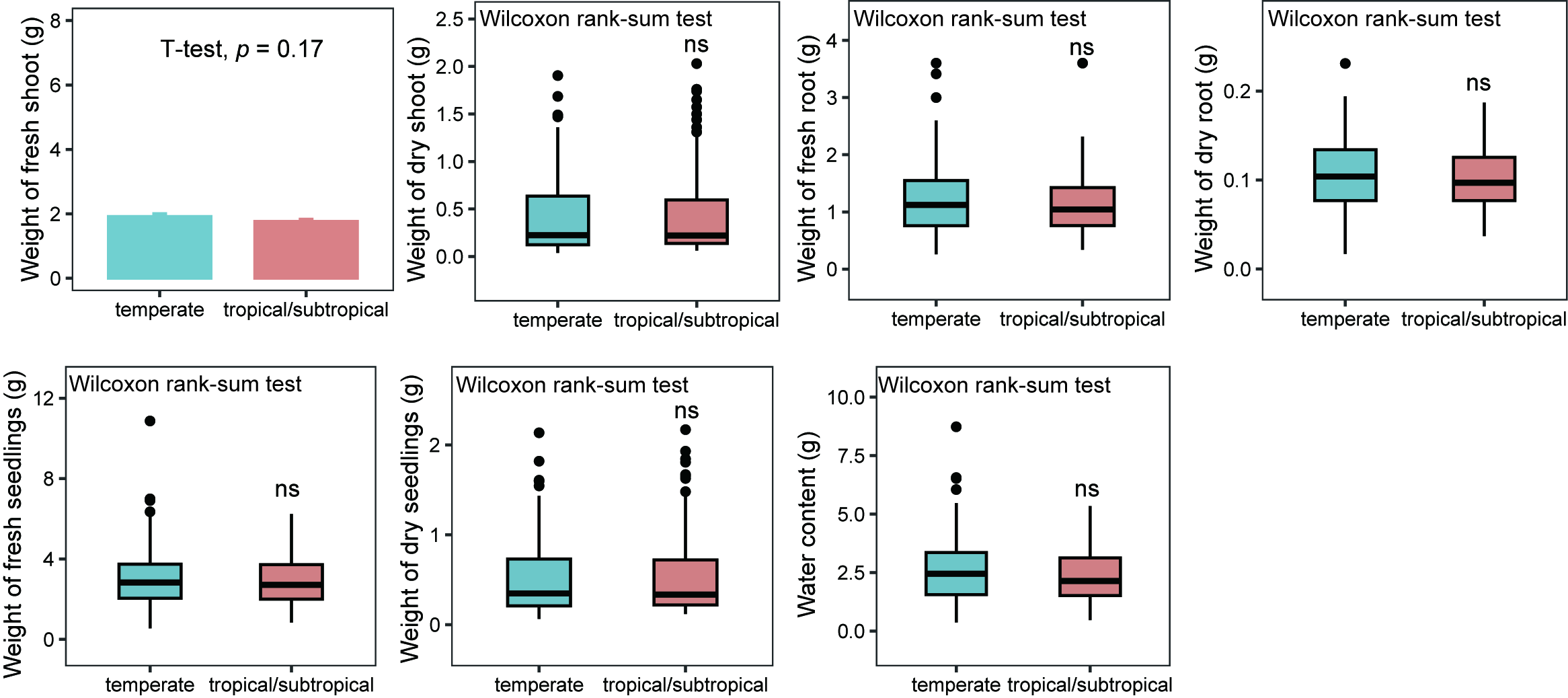


**Figure S2. Comparison of 7 W-traits between tropical/subtropical and temperate maize inbred lines.** The figure presents a series of violin plots comparing various weight-related traits, including weight of fresh shoot, dry shoot, total root, and other parameters, between tropical/subtropical (in orange) and temperate (in blue) maize inbred lines. Statistical significance for each trait is indicated by the *p*-value from the T-test or Wilcoxon rank-sum test.

**
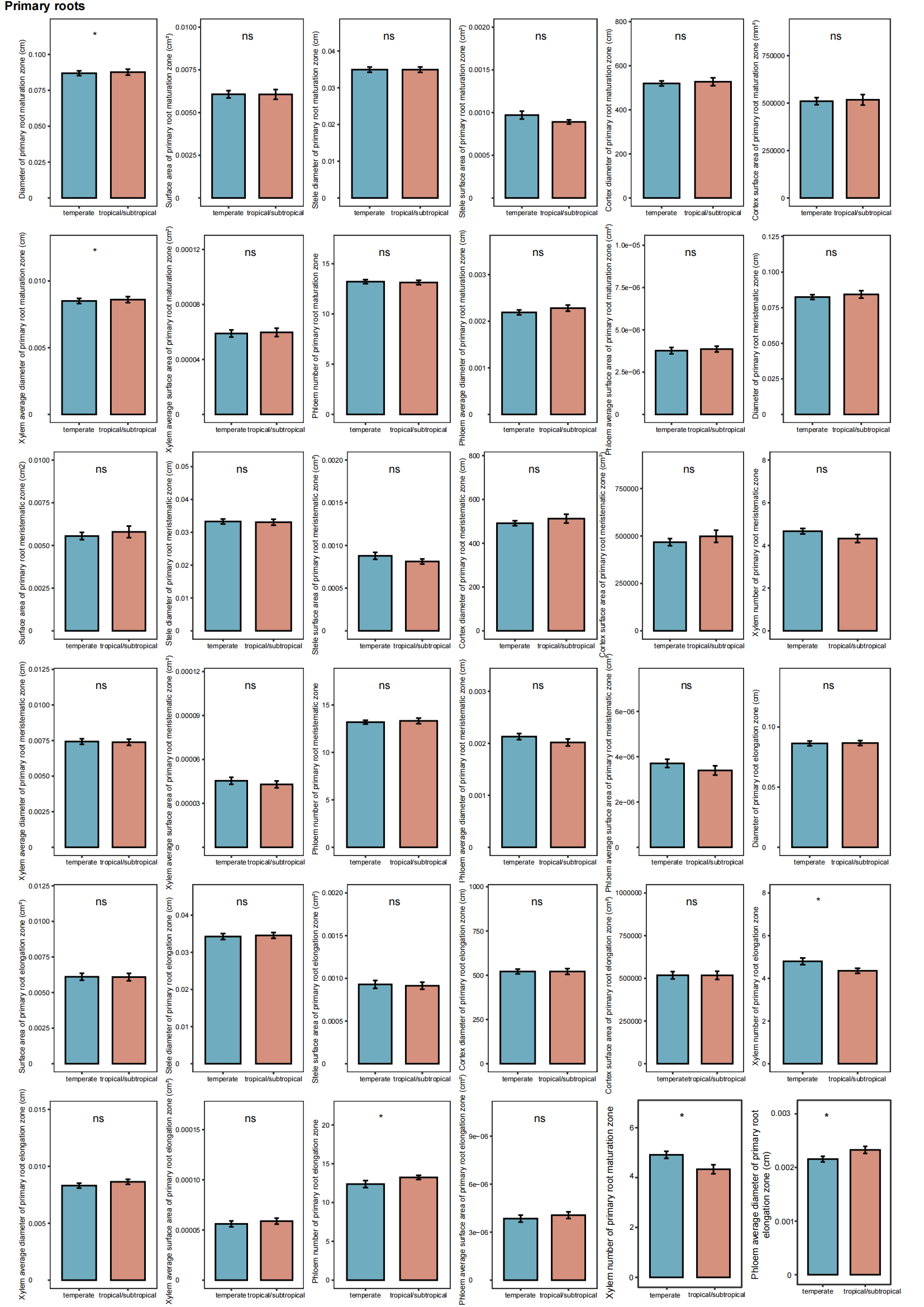
**

**Figure S3. Comparison of 36 S-traits in primary roots between tropical/subtropical and temperate maize inbred lines.** Statistical significance for each trait is indicated by the *p*-value from the T-test. The red asterisk represents a significant difference in this s-trait between tropical/subtropical and temperate lines.

**
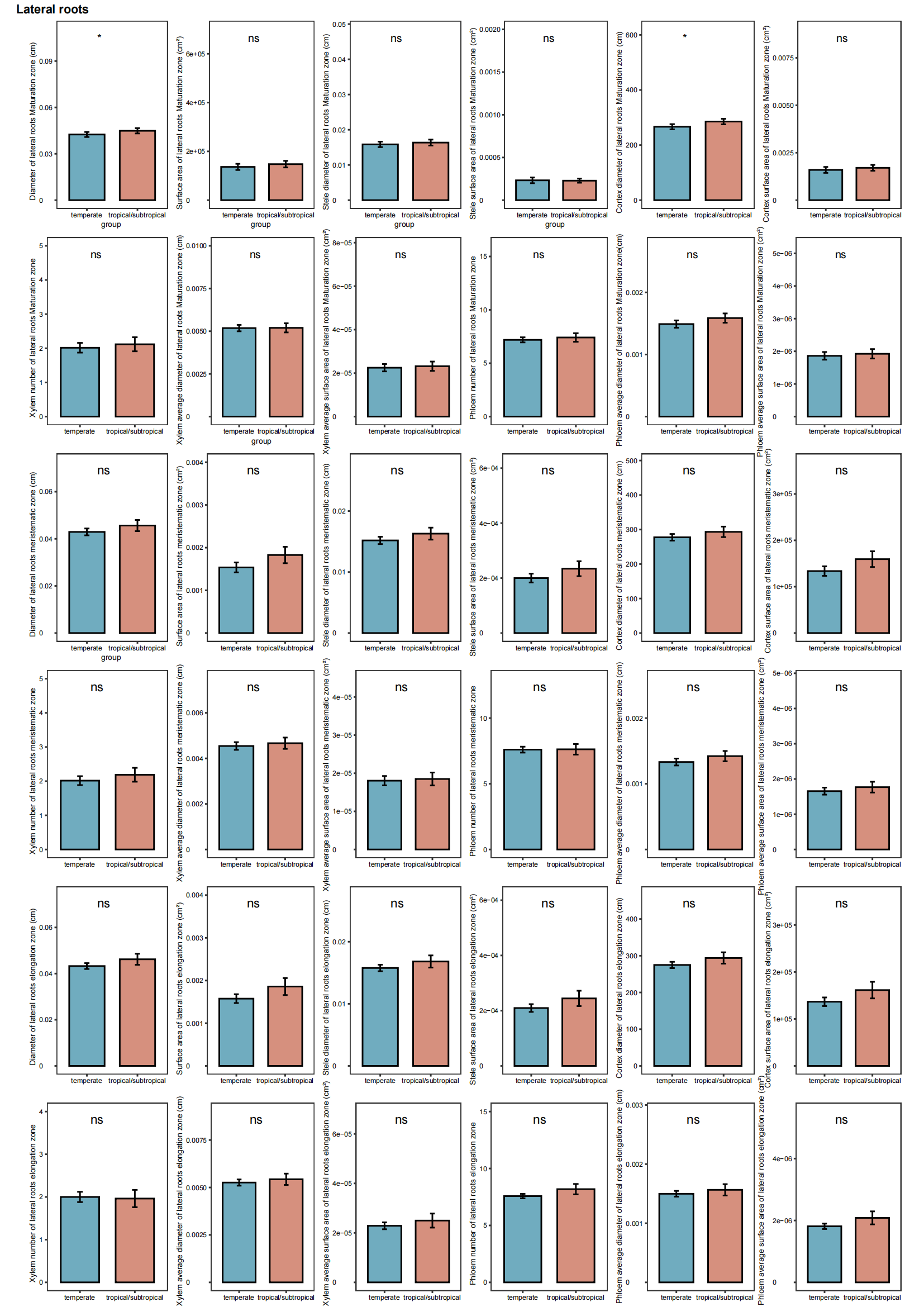
**

**Figure S4. Comparison of 36 S-traits in lateral roots between tropical/subtropical and temperate maize inbred lines.** Statistical significance for each trait is indicated by the *p*-value from the T-test. The red asterisk represents a significant difference in this s-trait between tropical/subtropical and temperate lines.


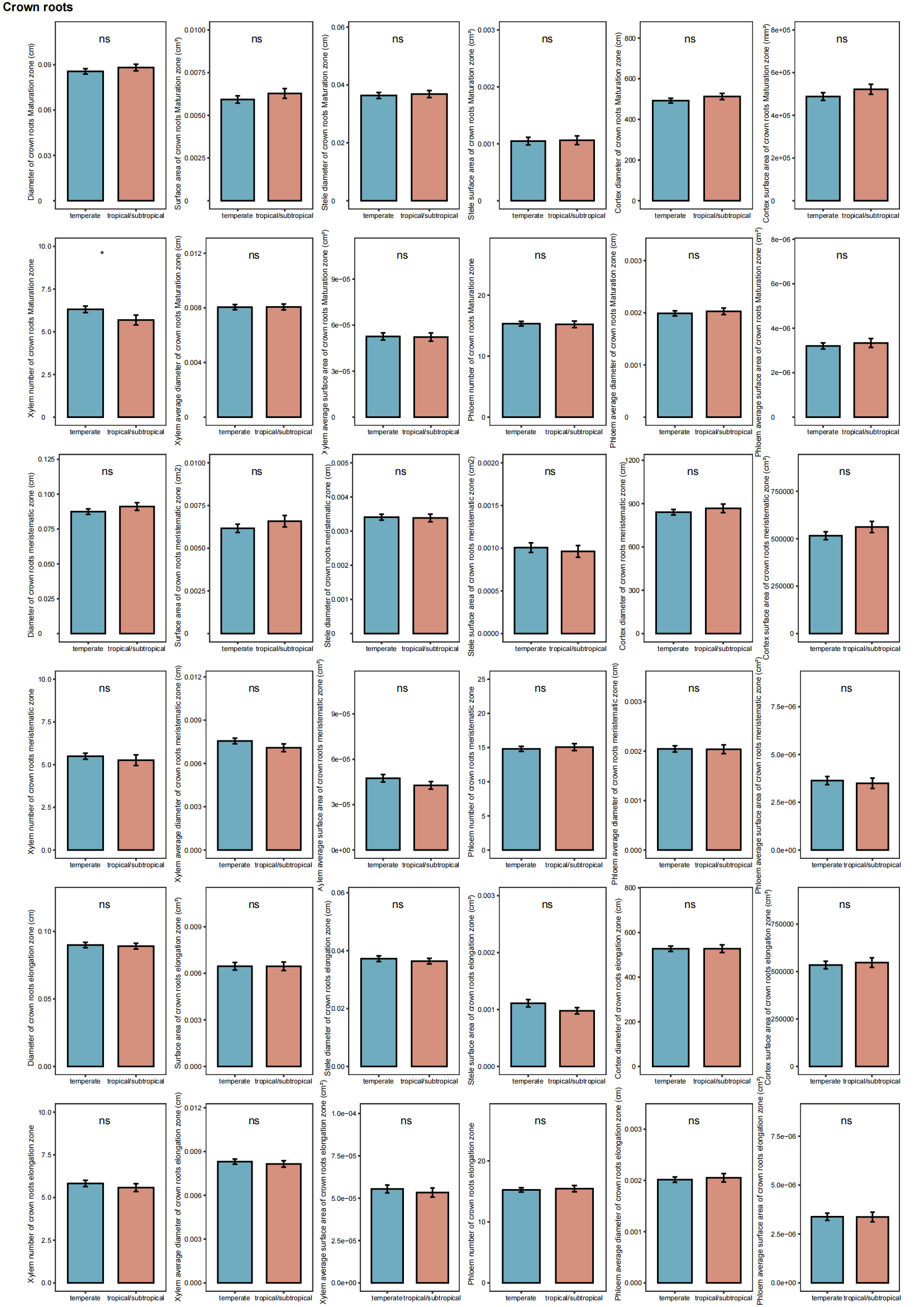


**Figure S5. Comparison of 36 S-traits in crown roots between tropical/subtropical and temperate maize inbred lines.** Statistical significance for each trait is indicated by the *p*-value from the T-test. The red asterisk represents a significant difference in this s-trait between tropical/subtropical and temperate lines.


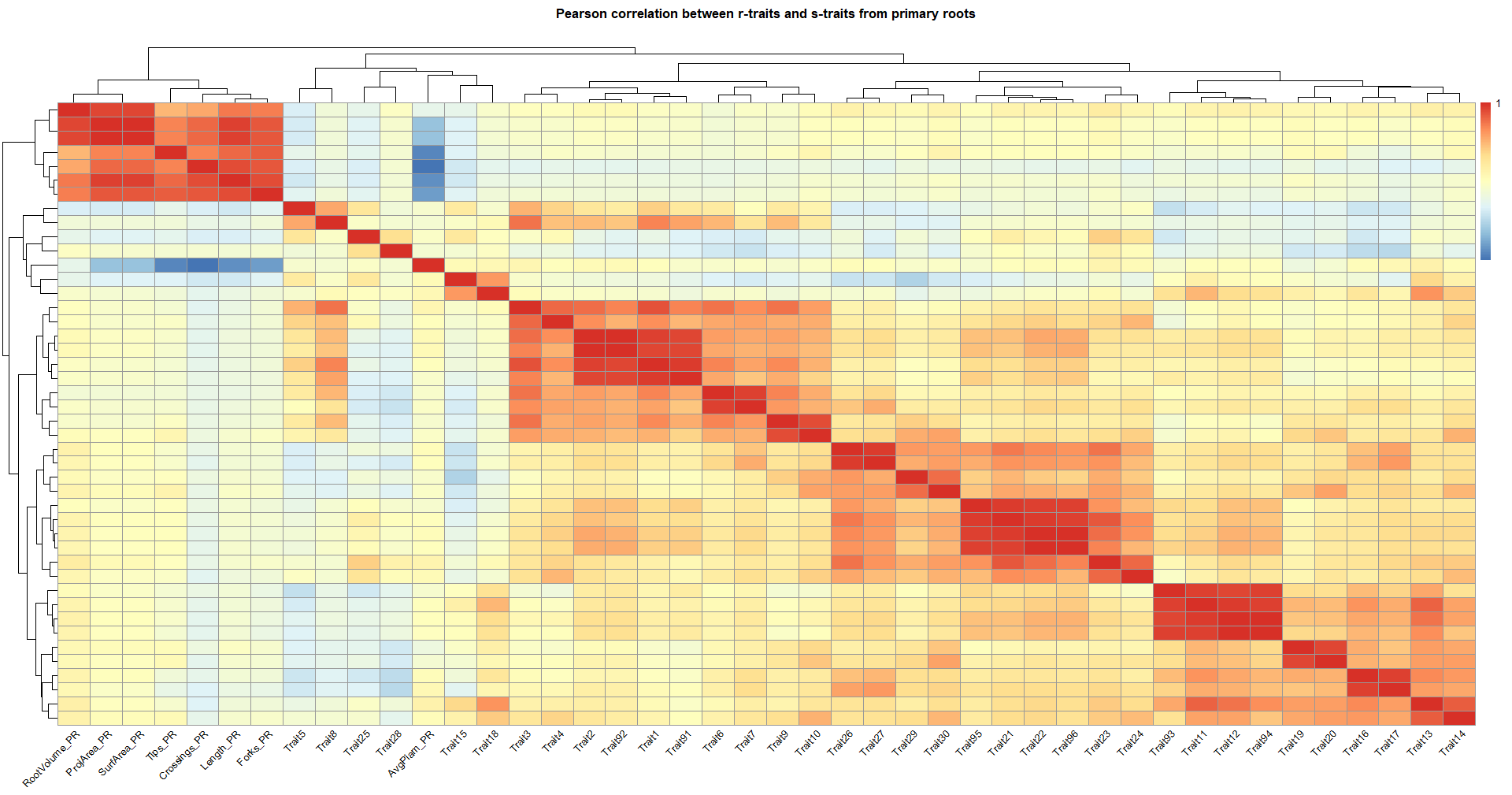


**Figure S6. The relevance of the R-traits and S-traits in primary roots.** Heatmap displaying the correlation analysis between 8 R-traits and 36 S-traits in primary roots.


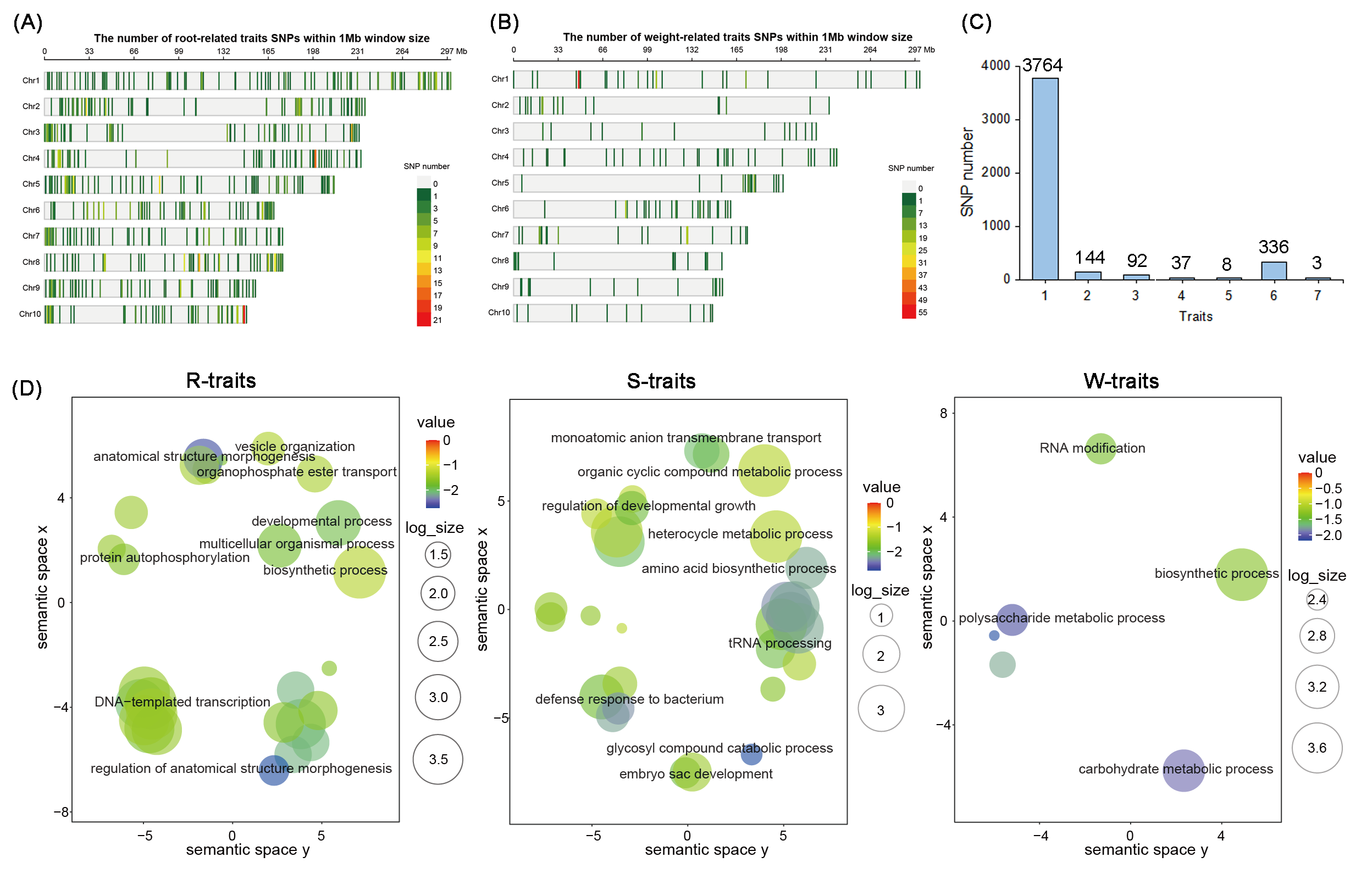


**Figure S7. GWAS identification of candidate genes for variation in W-traits and R-traits, in maize.** (A-B) Chromosomal distribution of loci associated with R-traits (A) and W-traits (B). Each vertical line indicates a lead SNP. (C) The distribution of SNP numbers across different traits. (D) GO terms for genes identified by GWAS with R-traits (left), S-traits (middle), and W-traits (right).


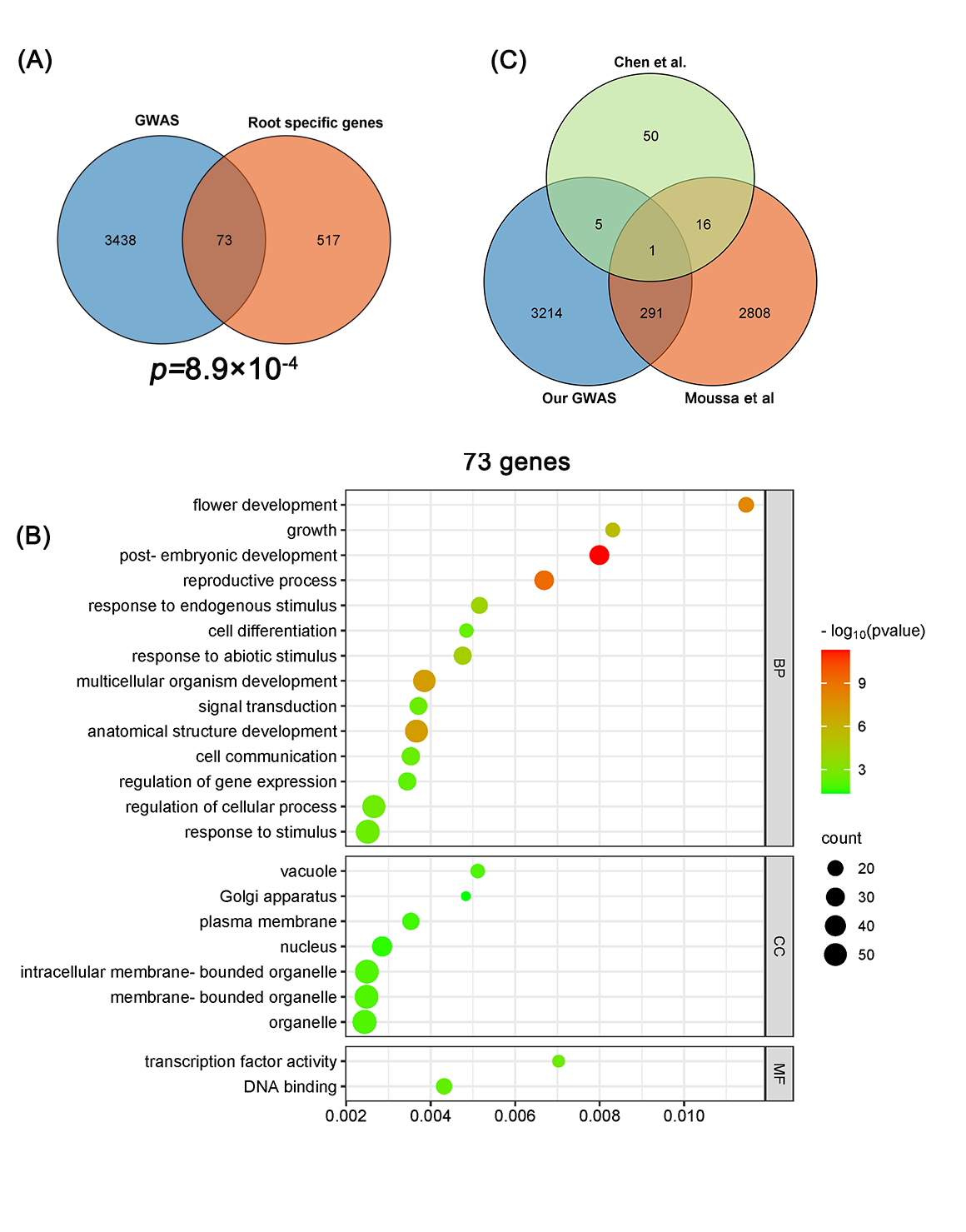


**Figure S8. Comparisons of candidate genes with other GWAS results in maize roots.** (A) A Venn diagram displays the unique and shared genes between the candidate genes and the root specific genes summarized by the previous study ^32^. (B) GO terms for 73 common genes in (A). (C) A Venn diagram displays the comparisons of candidate genes with other GWAS results in maize roots.


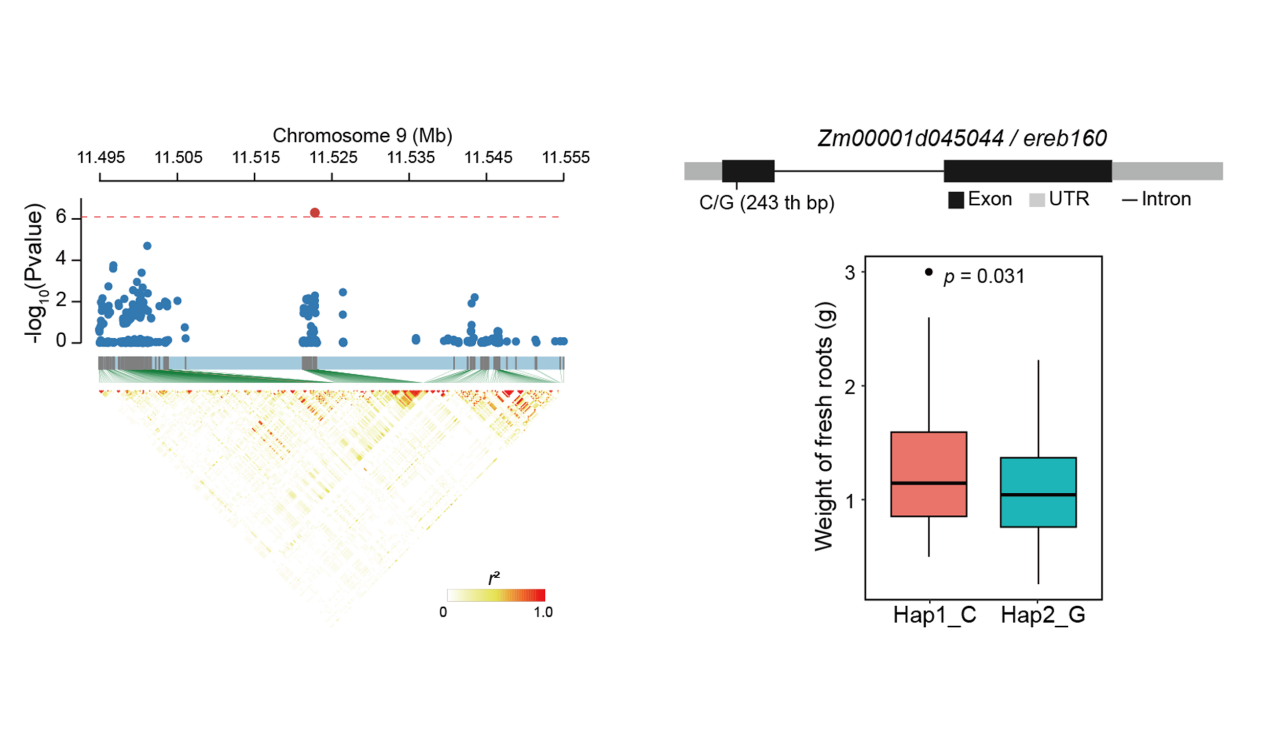


**Figure S9. Local Manhattan plot and LD statistic *r*^2^ values (left) for the *ereb160* gene associated with a W-trait, weight of fresh roots.** The upper right panel displays the gene structure of *ereb160*. The distributions of weight of fresh roots for the two groups based on the SNP chr9.S_11532641 are shown in the boxplot.
